# XMD8-92 and JWG-045 exhibit anti-ferroptotic activities, independently of inhibiting ERK5

**DOI:** 10.1038/s41598-026-42079-w

**Published:** 2026-02-28

**Authors:** Wei Zhang, Karmern Kan, Aidan B. Pidd, Gala Konteva, Weitao Xiao, Adam J. Pearson, Zejia Song, Qiuping Xu, Sam Butterworth, Alan J. Whitmarsh, Cathy Tournier

**Affiliations:** 1 Division of Cancer Sciences, School of Medical Sciences, Faculty of Biology, Medicine and Health (FBMH), University of Manchester, Michael Smith Building, Oxford Road, Manchester, M13 9PT UK; 2 Turku Bioscience Centre, University of Turku and Åbo Akademi University, Turku, Finland; 3 Division of Pharmacy and Optometry, School of Health Sciences, FBMH, University of Manchester, Manchester, UK; 4 Institute of Biomedicine, University of Turku, Turku, 20520 Finland; 5 Guangdong Provincial Key Laboratory of Malignant Tumor Epigenetics and Gene Regulation, Guangdong-Hong Kong Joint Laboratory for RNA medicine, Sun Yat-sen Memorial Hospital, Sun Yat-sen University, Guangzhou, China; 6 Division of Molecular and Cellular Function, School of Biological Sciences, FBMH, University of Manchester, Manchester, UK

**Keywords:** Biochemistry, Cancer, Cell biology, Drug discovery, Molecular biology, Oncology

## Abstract

Extracellular-regulated protein kinase 5 (ERK5) is an emerging therapeutic target in cancer, and small-molecule ERK5 inhibitors have been widely employed to define its role in tumour biology. Here, we show that the commonly used ERK5 inhibitors XMD8-92 and JWG-045 suppress RSL3-induced ferroptosis in breast cancer cells, in contrast to the next-generation ERK5 inhibitors JWG-071 and BAY-885, and the MEK5 inhibitor BIX02189. Using CRISPR-mediated gene editing, we generated ERK5-deficient breast cancer cells and found that XMD8-92 and JWG-045 retained their anti-ferroptotic activity against RSL3 in the absence of ERK5 expression, indicating clear off-target effects. Pathway-level analysis of bulk RNA-sequencing data using FerrDb-curated gene sets revealed no global alteration in ferroptotic activity in BT474 cells following XMD8-92 treatment. Interestingly, XMD8-92 did not inhibit RSL3-induced lipid peroxidation and preserved cell viability even after RSL3-induced ferroptosis initiation. Based on these observations, we propose that XMD8-92 confers transient resistance to ferroptotic cell death by maintaining plasma membrane integrity, potentially through enhanced membrane repair mechanisms. Collectively, these findings reveal a previously unrecognised off-target anti-ferroptotic activity of XMD8-92 and JWG-045, further highlighting the limitation of these compounds for ERK5-specific mechanistic studies.

**Supplementary Information:**

The online version contains supplementary material available at 10.1038/s41598-026-42079-w.

## Introduction

The extracellular-regulated protein kinase 5 (ERK5), encoded by the mitogen-activated protein kinase 7 (*MAPK7*) gene, is a unique member of the MAPK family. Activated via the MAPK/ERK kinase kinases (MEKK) 2/3-MEK5 pathway, ERK5 stands out from the rest of the MAPK family due to its extended C-terminal tail which comprises structural domains involved in controlling nuclear translocation and ERK5-mediated transcriptional regulation through myocyte enhancer factor 2 (MEF2) family members^1^. Soon after its discovery, studies showed that ERK5 signalling played a role in key cancer hallmarks and promoted resistance to targeted agents^2^. Furthermore, while MEK5 or ERK5 mutations are rare in cancer, patients with tumours displaying aberrant MEK5 and/or ERK5 expression and activation exhibit significantly worse clinical outcomes. For instance, increased ERK5 expression and/or activation levels are consistently associated with the acquisition of more aggressive tumour phenotypes across different molecular subtypes of breast cancer^3,4^. In line with these observations, we found that ERK5 depletion reduced the metastatic activity of triple negative breast cancer cells^5^. Additionally, pharmacological inhibition of ERK5 overcame HER2-targeted resistance in HER2 + breast cancer cells^6^.

To better understand how ERK5 signalling contributes to cancer, we analysed the proteins that interacted with ERK5 in HeLa cells. This led us to identify glutathione peroxidase 4 (GPX4) as one of its binding partners^7^. GPX4 is an enzyme that protects cells from oxidative stress by utilising glutathione (GSH) to neutralise peroxides^8^. Of particular interest, inhibition of GPX4 by compounds such as RSL3, ML162, or ML210 leads to excessive lipid peroxidation triggering ferroptosis, an iron-dependent form of cell death characterised by membrane damage^9–12^. This distinctive mode of cell death represents a promising strategy to eliminate cancer cells that have developed resistance to conventional therapies. Notably, drug-induced persistent, mesenchymal and de-differentiated cancer cells that are typically resistant to apoptosis, are particularly susceptible to ferroptosis^13–15^. These observations prompted us to explore whether ERK5 signalling might influence how breast cancer cells respond to ferroptosis-inducing treatments.

Like other MAPKs, ERK5 mainly acts through its kinase activity. Consequently, numerous studies have taken advantage of the availability of small molecule inhibitors to explore the functions of ERK5 signalling in cancer cells (Supplementary Fig. S1 online)^16^. XMD8-92 was the first compound of the XMD series exhibiting selectivity for ERK5 and showing promising anti-proliferative and anti-inflammatory effects^17–23^. However, this inhibitor was later found to have off-target effects on bromo-domain containing protein 4 (BRD4), doublecortin-like kinases (DCLKs), and leucine-rich repeat kinase 2 (LRRK2), which casted doubt on whether tumour cells were actually dependent on ERK5 activity^17,18,24–27^. The development of AX15836, a XMD8-92 analogue without BRD4 inhibitory activity, did not resolve this issue because it paradoxically activates ERK5 transcriptional activity by inducing a conformational change in the kinase domain leading to the exposure of the C-terminal nuclear localisation signal^25,28^. Optimisations to enhance the specificity of XMD8-92 produced two new compounds, namely JWG-045 and JWG-071, which display increased selectivity for ERK5 over BRD4, but retain an inhibitory activity towards LRRK2 and DCLK^24,29^. The most recently developed compound, BAY-885, was identified through high-throughput screening and appears to be the most specific ERK5 inhibitor^30^. All these ERK5 inhibitors act in an ATP-competitive manner.

Based on this knowledge we sought to utilise a variety of ERK5 inhibitors to dissect the contribution that ERK5 played in mediating ferroptotic death in breast cancer.

## Results

### Cancer cells with elevated ERK5 expression exhibit hypersensitivity to ferroptosis

**Fig. 1:**
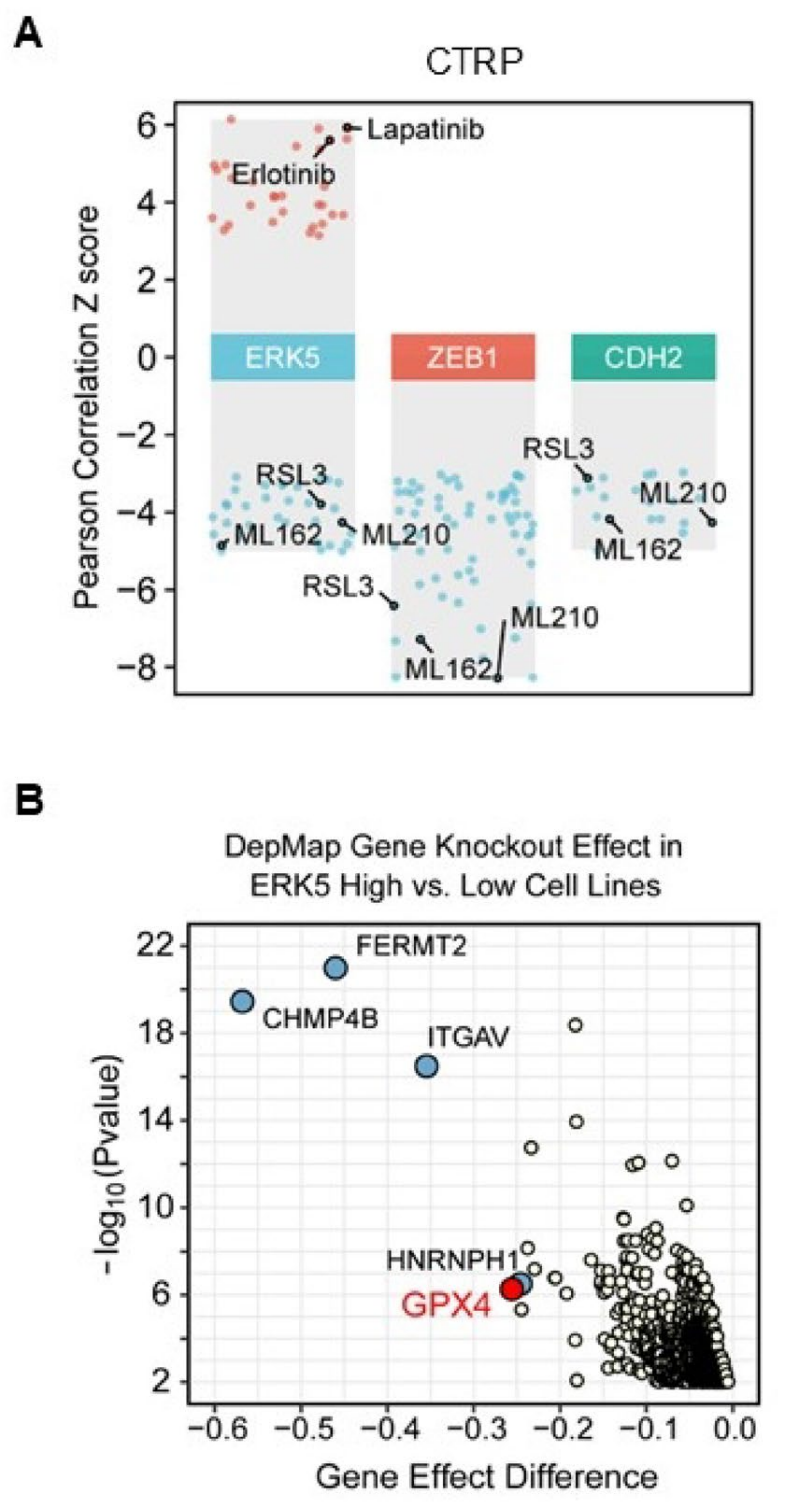
High *ERK5* mRNA expression inversely correlates with ferroptotic sensitivity. (**A**) *ERK5*,* ZEB1* and *CDH2* mRNA expression in 860 cancer cell lines and correlation with their response to 481 compounds were curated from the CTRP portal. Z-scores represent standardised Pearson correlation coefficients between mRNA abundance (log₂ TPM from cancer cell line encyclopaedia RNA-seq) and the viability profiles of cancer cell lines in response to each drug across the DepMap dataset. Negative Z-scores indicate that high expression of ERK5, ZEB1 or CDH2 is associated with increased drug sensitivity, while positive Z-scores indicate relative resistance. Cut-off was set as 3 and − 3. (**B**) Cancer cell lines were ranked by *ERK5* mRNA expression (log₂ TPM) and divided into the top 30% (ERK5-high) and bottom 30% (ERK5-low) cohorts. For each gene, gene effect scores downloaded from the DepMap portal, where lower values indicate greater dependency, were compared between groups. Gene effect difference was defined as the difference in median gene effect scores between ERK5-high and ERK5-low cell lines. Statistical significance was assessed using the Wilcoxon rank-sum test, and p-values were transformed to –log_10_ for visualisation.

To gain a broad view into how ERK5 signalling influenced therapeutic responses, we correlated the *ERK5* (also known as *MAPK7*) transcript level with the drug sensitivity dataset generated by the Cancer Therapeutics Response Portal (CTRP). This dataset consists of 860 cancer cell lines treated with 481 small-molecule probes and drugs, referred to as the ‘Informer Set’ compounds. Consistent with our previous demonstration, and that of others, that ERK5 signalling mediated the resistance of breast and lung cancer cells to HER-targeted therapies^6,31^, elevated *ERK5* mRNA expression negatively correlated with cancer cell sensitivity to lapatinib and erlotinib (Fig. 1A). Conversely, cells exhibiting a high level of *ERK5* mRNA appeared to be the most sensitive to GPX4 inhibitors, i.e. RSL3, ML162 and ML210 (Fig. 1A). The same was observed for cells expressing transcripts encoding the mesenchymal markers *ZEB1* or *CDH2*, consistent with evidence that mesenchymal cancer cells are more sensitive to ferroptosis compared with cancer cells displaying an epithelial phenotype^15^. In parallel, we analysed whole-genome CRISPR gene knock-out effects in human cancer cell lines with high and low *ERK5* transcript expression (top 30% vs. bottom 30%) from the Cancer Dependency Map (DepMap) portal. The data further demonstrated that cell lines with an elevated level of *ERK5* mRNA were highly susceptible to targeted deletion of *GPX4* (Fig. 1B). This observation was of particular interest given that our prior quantitative proteomic analysis had identified GPX4 as a binding partner of ERK5^7^.

### XMD8-92 and JWG-045 prevent RSL3-induced ferroptotic death

**Fig. 2:**
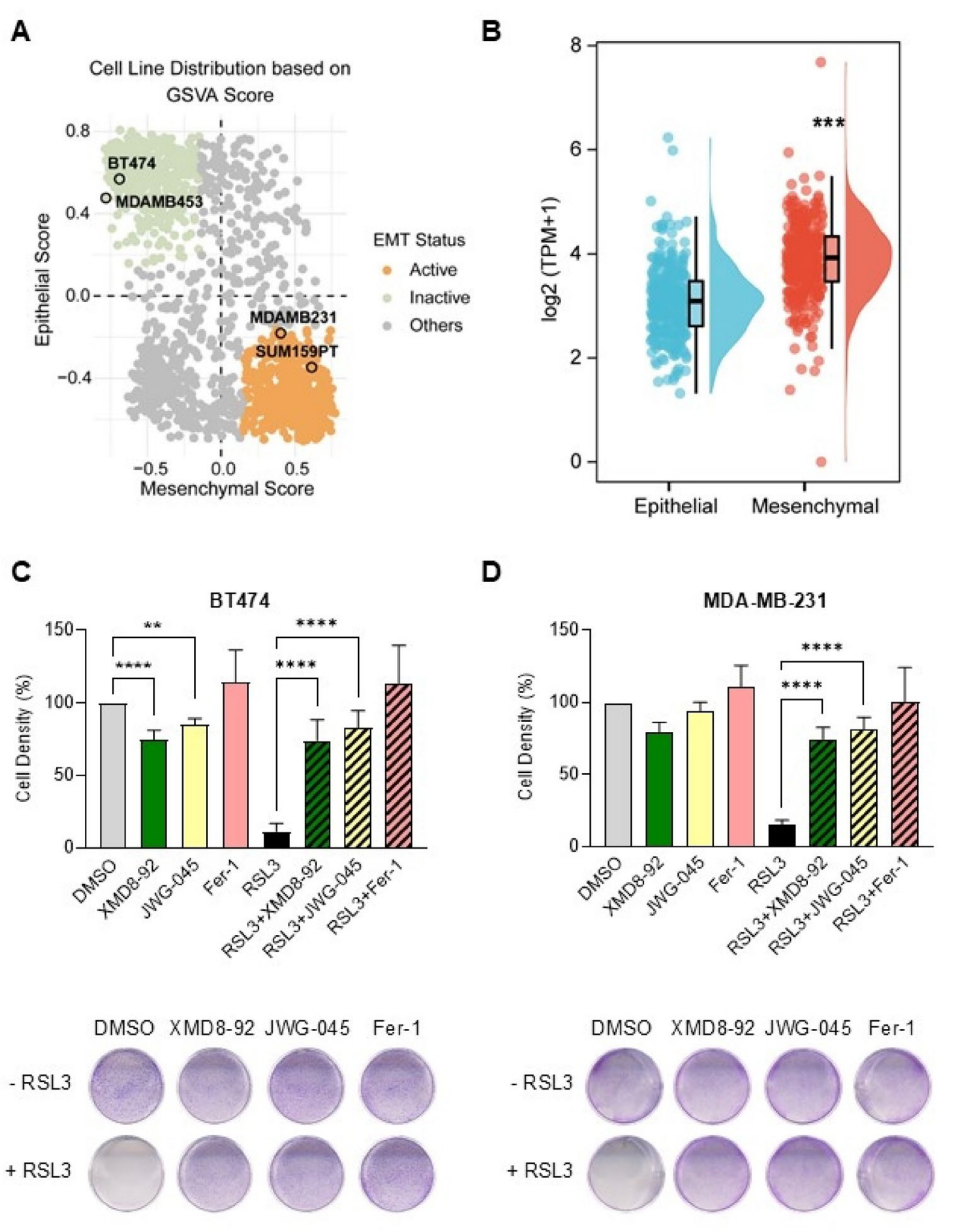
XMD8-92 and JWG-045 exhibit an anti-ferroptotic activity. (**A**) RNA-sequencing datasets from DepMap were utilised to subdivide human cancer cell lines into epithelial-like and mesenchymal-like groups based on GSVA score established from epithelial and mesenchymal gene signatures. HER2-positive breast cancer BT474 and MDA-MB-453 cell lines were highlighted as epithelial-like cells, whilst triple negative breast cancer MDA-MB-231 and SUM159PT cells displayed a mesenchymal phenotype. (**B**) Levels of expression of *ERK5* transcript in epithelial-like and mesenchymal-like human cancer cell lines (mean ± SD). ***p ≤ 0.001 (by unpaired t-test). (**C** and **D**) Quantification of cell density by crystal violet staining of BT474 and MDA-MB-231 cell lines treated with XMD8-92 (5 µM), JWG-045 (3 µM), Ferrostatin-1 (Fer-1, 1 µM) or RSL3 (250 nM), alone or in combination, for 24 h. Mock treated cells with DMSO were used as controls. The data are expressed as the mean percentage of cell density ± SD (*n* = 4). One-way ANOVA was performed for statistical analyses.

To assess the requirement of ERK5 in mediating ferroptosis, we tested the effect of XMD8-92 and JWG-045 on the response of breast cancer cells to RSL3. BT474 cells, a model of luminal B (ER+/PR+/HER2+) cancer subtype, and the triple-negative breast cancer (TNBC) cell line MDA-MB-231 were employed to represent epithelial and mesenchymal breast cancer phenotypes, respectively, as shown by gene set variation analysis (GSVA) (Fig. 2A). Interestingly, we observed that the level of *ERK5* transcript was significantly higher in the mesenchymal cells compared to epithelial cells (Fig. 2B), which aligns with our previous findings showing that MDA-MB-231 cells express more ERK5 protein than BT474 cells^5,6^. Consistent with the high sensitivity of BT474 cells to ERK5 inhibition^6^, treatment with either XMD8-92 or JWG-045 alone resulted in a modest, but statistically significant, reduction in BT474 cell density after 24 h (Fig. 2C). More remarkably, the inhibitors reduced the cytotoxicity of RSL3 in both cell lines (Fig. 2C and D). The protective effect of Ferrostatin-1 (Fer-1) against RSL3 confirmed that RSL3-induced cell death occurred via ferroptosis (Fig. 2C and D).

### Alternative inhibitors of ERK5 signalling fail to protect breast cancer cells from ferroptotic death

Given the known ERK5-independent off target effects of XMD8-92 and JWG-045^17,25^, we tested the activity of two additional ERK5 inhibitors, namely JWG-071 and BAY-885, alongside that of BIX02189, an inhibitor of MEK5^32^. Like XMD8-92 and JWG-045, JWG-071, BAY-885 and BIX0289 suppressed ERK5 phosphorylation in BT474 cells under basal conditions and in EGF-stimulated MDA-MB-231 cells, as demonstrated by the loss of the mobility shift (Supplementary Fig. S2A and B online). We previously found that ERK5 inhibition by JWG-045 enhanced the anti-proliferative activity of HER2 inhibitors in lapatinib- or trastuzumab-resistant BT474 cell lines^6^. Consistently, we observed that JWG-071 restored the growth inhibitory effect of trastuzumab in BT474-resistant cells to the level achieved by trastuzumab monotherapy in the parental BT474 cell line (Supplementary Fig. S2C online). Moreover, consistent with the exquisite sensitivity of HER2-expressing breast cancer cells to ERK5 inhibition by JWG-045^6^, JWG-071 significantly reduced BT474 cell density after 6 days, independently of their resistance status to trastuzumab (Supplementary Fig. S2C online). Interestingly, the effect of JWG-071 as a single treatment was noticeably stronger than that of JWG-045 alone. Overall, this experiment demonstrated that JWG-071 and JWG-045 exhibited similar inhibitory activity in BT474 cells after 6 days.

**Fig. 3:**
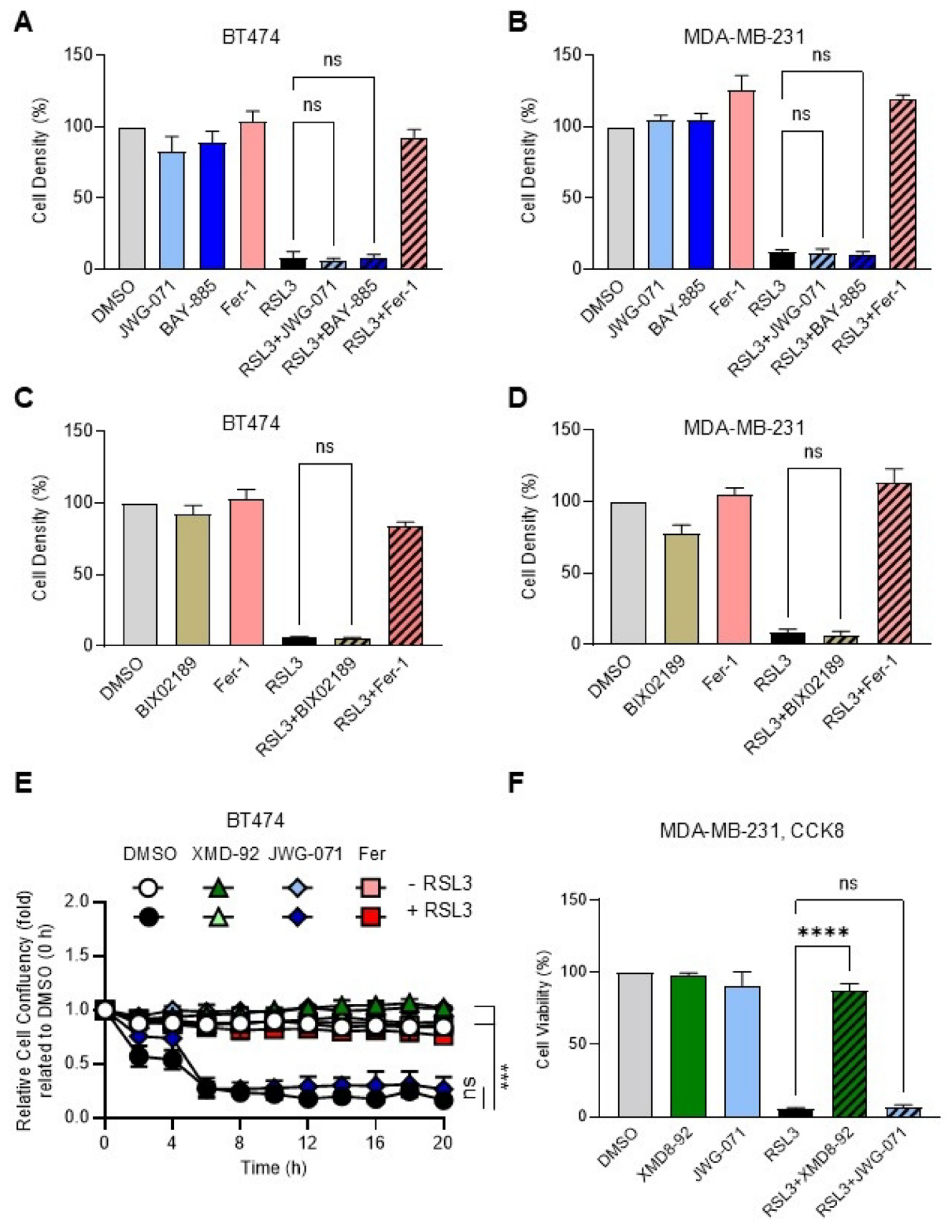
Targeting ERK5 signalling by JWG-071, BAY-885 or BIX02189 does not protect breast cancer cells from ferroptotic death. BT474 (A, C and E) and MDA-MB-231 cells (B, D and F) were treated with XMD8-92 (5 µM), JWG-071 (3 µM), BAY-885 (5 µM), BIX02189 (5 µM), Ferrostatin-1 (Fer-1, 1 µM), or RSL3 (250 nM), as single agents or in combination, for 24 h unless indicated otherwise. Mock treated cells with DMSO were used as controls. (**A**–**D**) Cell density was quantified by crystal violet staining. The data are expressed as the mean percentage of cell density ± SD (*n* = 3). (**E**) BT474 cells were monitored in the IncuCyte SX5 (Sartorius) using a phase-contrast camera, and cell confluency was quantified over a 20-hour period. Images were acquired every 2 h. The data are expressed as the mean fold of cell confluency ± SD (*n* = 3). (**F**) Cell viability was quantified by CCK8 assay. The data are expressed as the mean percentage of cell viability ± SD (*n* = 3). One-way ANOVA was performed for statistical analyses to compare the effect of RSL3 versus RSL3 combined with ERK5 inhibitor treatments.

However, a 24-hour incubation with JWG-071 or BAY-885 had no effect on the growth of BT474 or MDA-MB-231 cells and did not protect cells against RSL3-induced ferroptosis (Fig. 3A and B). The same discrepancy between XMD8-92 or JWG045, and JWG-071 or BAY-885 was observed in response to ML210 (Supplementary Fig. S3A online). Likewise, pre-incubation of BT474 or MDA-MB-231 cells with BIX0289 did not block RSL3-induced cytotoxicity (Fig. 3C and D). To confirm the differential protective effects of ERK5 inhibitors against ferroptosis, we performed live-cell imaging using IncuCyte (Fig. 3E). Consistent with our previous results, JWG-071 failed to inhibit the time-dependent decrease in BT474 cell confluency, which was maximal after 5 h of incubation with RSL3. In contrast, XMD8-92 restored the growth of RSL3-treated BT474 cells to a level comparable to those of cells treated with DMSO alone or with RSL3 in combination with Ferrostatin-1 (Fer-1). Similarly, whereas JWG-071 had no protective effect, XMD8-92 maintained the viability of MDA-MB-231 cells incubated with RSL3 (Fig. 3F). We further demonstrated that incubation of BT474 cells with two different BRD4 inhibitors, namely JQ1 and Birabresib, alone or in combination with JWG-071, did not reproduce the inhibitory effect of XMD8-92 and JWG-045 on RSL3-induced cytotoxicity (Supplementary Fig. S3B online). This suggested that the activity of XMD8-92 and JWG-045 on ferroptosis was unlikely to involve the blockade of BRD4.

### The anti-ferroptotic activity of XMD8-92 and JWG-045 in cancer cells is independent of ERK5

**Fig. 4:**
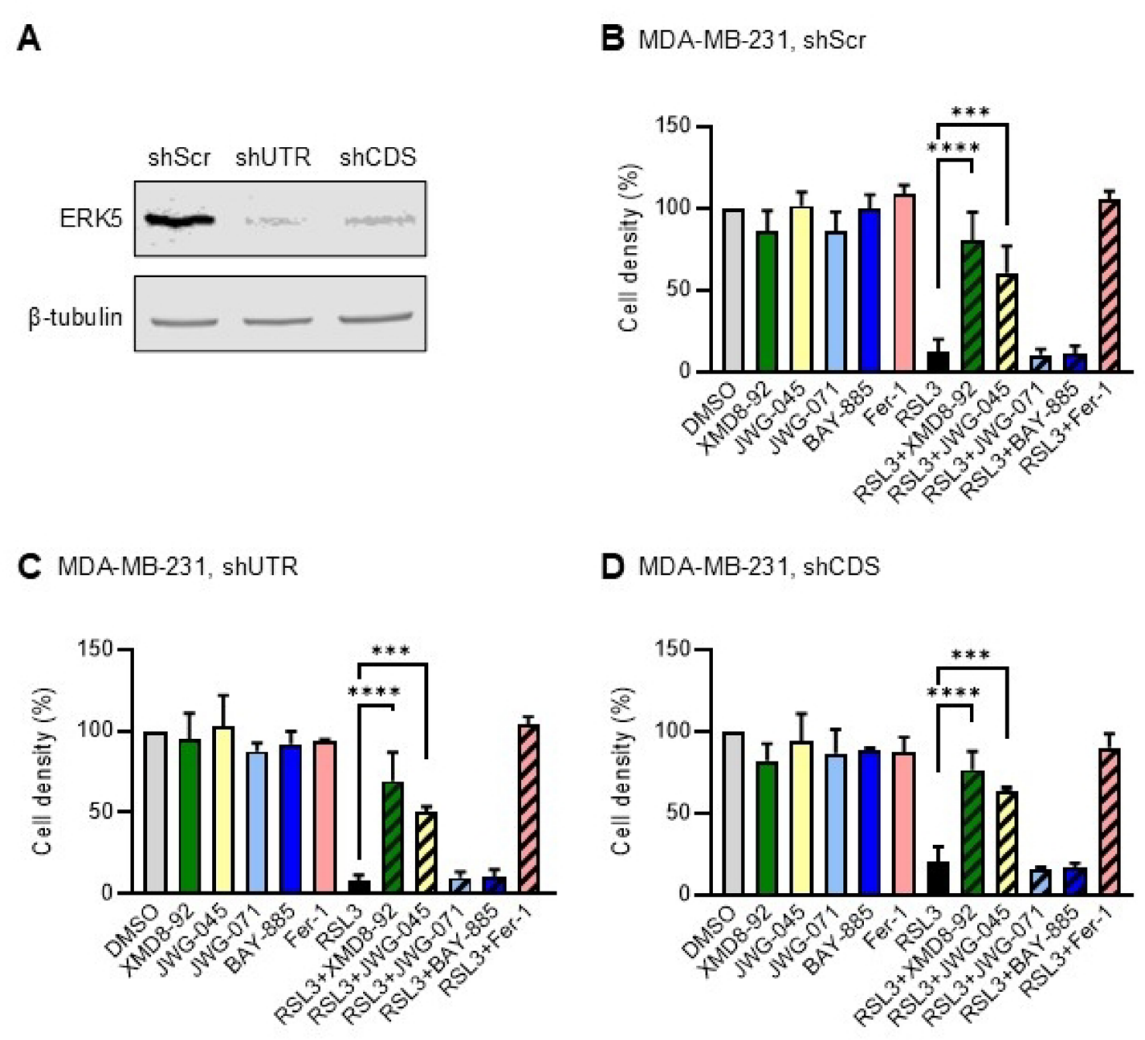
Effect of ERK5 inhibitors on RSL3-induced ferroptosis in ERK5 silenced MDA-MB-231 cells. (**A**) Immunoblot analysis of ERK5 expression in control (shScr) and ERK5 silenced MDA-MB-231 cells via stable expression of shERK5(UTR) and shERK5(CDS). β-tubulin expression was used as loading control. Similar results were obtained in two independent experiments. (**B**–**D**) Control and ERK5 silenced MDA-MB-231 cells were treated with XMD8-92 (5 µM), JWG-045 (3 µM), JWG-071 (3 µM), BAY-885 (5 µM), Ferrostatin-1 (Fer-1, 1 µM), or RSL3 (250 nM), as single agents or in combination, for 24 h. Mock treated cells with DMSO were used as controls. Cell density was quantified by crystal violet staining. One-way ANOVA was utilised to analyse statistical differences between mean percentages of cell density ± SD (*n* = 3).

To further analyse the effect of XMD8-92 and JWG-045 on ferroptosis, we took advantage of two distinct ERK5 knockdown MDA-MB-231 cell lines engineered in our laboratory through stably transfecting two different shRNA targeting the 3’UTR or the coding sequence (CDS) of *ERK5*^5^. We confirmed that both shRNAs effectively reduced ERK5 expression compared to the scrambled RNA control (shScr) (Fig. 4A). As previously demonstrated, whereas JWG-071 and BAY-885 had no effect, XMD8-92 and JWG-045 blocked RSL3-induced ferroptotic death in shScr-expressing MDA-MB-231 cells (Fig. 4B). More importantly, the anti-ferroptotic activity of XMD8-92 and JWG-045 was observed in ERK5-silenced cells treated with RSL3 (Fig. 4C and D).

**Fig. 5:**
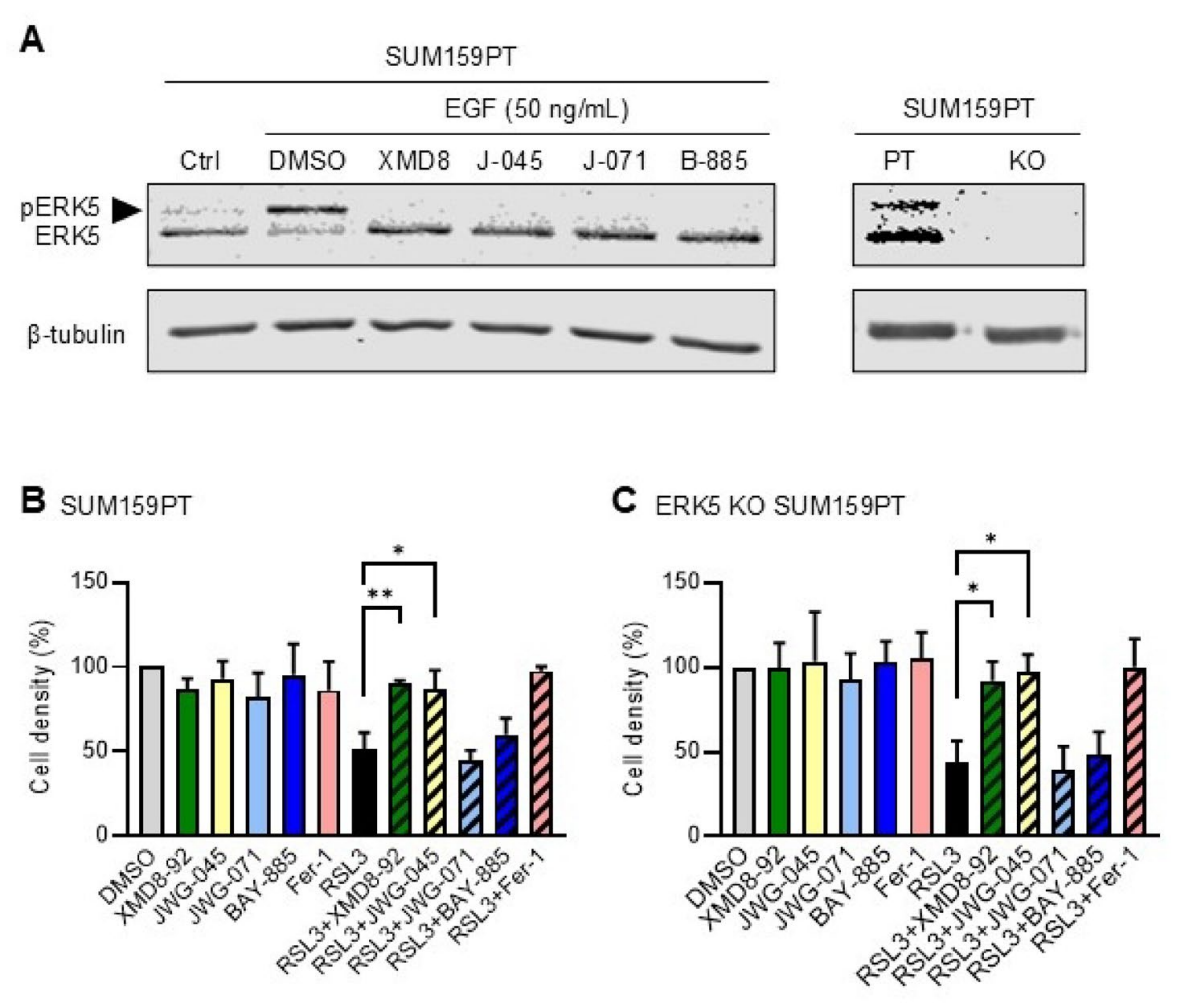
XMD8-92 and JWG-045 suppress RSL3-induced ferroptosis in ERK5 knock-out SUM159PT cells. (**A**) Immunoblot analysis of ERK5 expression in parental (PT) and ERK5 KO SUM159PT cells. Where indicated, parental cells were mock treated with DMSO or treated with XMD8-92 (XMD8; 5 µM), JWG-045 (J-045; 3 µM), JWG-071 (J-071; 3 µM) or BAY-885 (B-885; 5 µM) for 1 h, followed by EGF (10 ng/mL) stimulation for 10 min. β-tubulin expression was used as loading control. Similar results were obtained in two independent experiments. The arrow indicates phosphorylated ERK5. (**B** and **C**) Parental and ERK5 KO SUM159PT cells were treated with XMD8-92 (5 µM), JWG-045 (3 µM), JWG-071 (3 µM), BAY-885 (5 µM), Ferrostatin-1 (Fer-1, 1 µM), or RSL3 (250 nM), as single agents or in combination, for 24 h. Mock treated cells with DMSO were used as controls. Cell density was quantified by crystal violet staining. One-way ANOVA was utilised to analyse statistical differences between mean percentages of cell density ± SD (*n* = 3).

Next, we utilised CRISPR-mediated gene editing to inactivate the *ERK5* locus in breast cancer cells. Given that we were not able to culture MDA-MB-231 cells as single clones, this was achieved by employing another TNBC cell line, namely SUM159PT, derived from poorly differentiated primary breast cancer^33^. Unlike MDA-MB-231 cells, SUM159PT cells exhibited a small level of ERK5 phosphorylation under basal conditions (Fig. 5A). This was surprising given that ERK5 had never been reported to be constitutively phosphorylated in TNBC before. We confirmed the ability of ERK5 inhibitors to suppress ERK5 phosphorylation in parental SUM159PT cells under basal conditions and in response to EGF stimulation, as indicated by the loss of the mobility shift of the protein (Fig. 5A). Homozygous targeted deletion was demonstrated by the complete loss of ERK5 protein expression (Fig. 5A). In subsequent experiments, this cell line will be referred to as ERK5 knock-out (KO) SUM159PT cells. Consistent with our analysis of ERK5-silenced MDA-MB-231 cells (Fig. 4), whereas JWG-071 and BAY-885 had no effect, both XMD8-92 and JWG-045 inhibited the ferroptotic response of SUM159PT cells to RSL3, even in the absence of ERK5 (Fig. 5B,C).

**Fig. 6:**
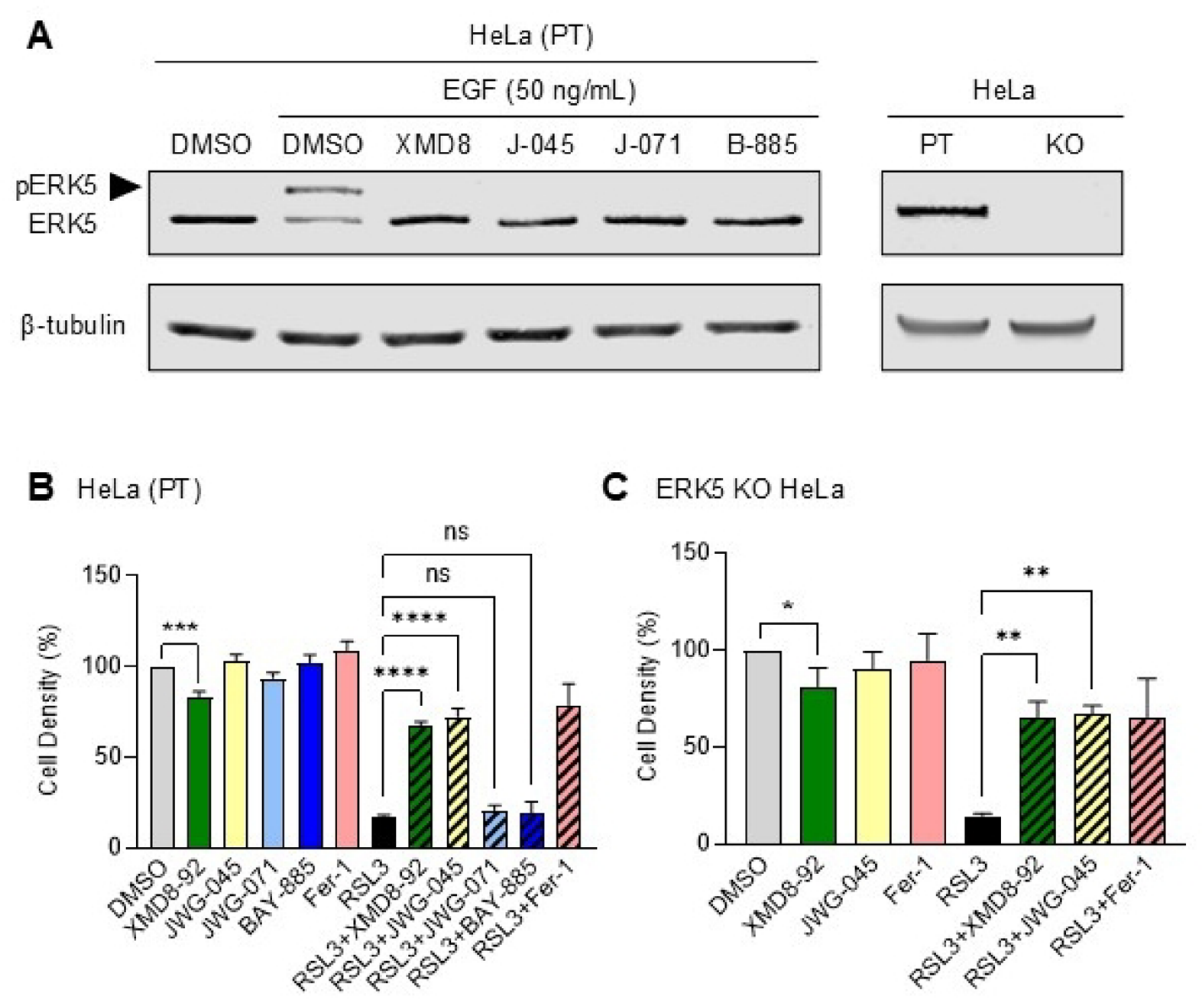
ERK5 is not required for mediating RSL3-induced ferroptosis in HeLa cells. (**A**) Immunoblot analysis of ERK5 expression in parental (PT) and ERK5 KO HeLa cells. Where indicated, parental cells were mock treated with DMSO or pre-treated with XMD8-92 (XMD8; 5 µM), JWG-045 (J-045; 3 µM), JWG-071 (J-071; 3 µM) or BAY-885 (B-885; 5 µM) for 1 h, followed by EGF (10 ng/mL) stimulation for 10 min. β-tubulin expression was used as loading control. Similar results were obtained in two independent experiments. The arrow indicates phosphorylated ERK5. (**B** and **C**) Parental and ERK5 KO HeLa cells were treated with XMD8-92 (5 µM), JWG-045 (3 µM), JWG-071 (3 µM), BAY-885 (5 µM), Ferrostatin-1 (Fer-1, 1 µM), or RSL3 (2500 nM), alone or in combination, for 24 h. Mock treated cells with DMSO were used as controls. Cell density was quantified by crystal violet staining. The data are expressed as the mean percentage of cell density ± SD (*n* = 3). One-way ANOVA was performed for statistical analyses.

We expanded this analytical approach to HeLa cells, a human-derived cervical cancer cell line model which had been employed for quantitative proteomic analysis of the ERK5 interactome which led to the demonstration that GPX4 was a binding partner of ERK5^7^. Initially, we established the conditions to inhibit EGF-mediated ERK5 activation by treating the cells with XMD8-92, JWG-045, JWG-071 and BAY-885 and engineered homozygous targeted deletion of *ERK5* in HeLa cells by CRISPR-mediated gene editing (Fig. 6A). XMD8-92 as a single treatment exerted a small suppressive effect on the growth of HeLa cells, independently of ERK5 (Fig. 6B and C). Moreover, like in breast cancer cell lines, XMD8-92 and JWG-045 were the only two inhibitors capable of protecting HeLa cells against RSL3-induced ferroptosis (Fig. 6B). The protective effect of XMD8-92 and JWG-045 persisted in ERK5 KO HeLa cells (Fig. 6C).

Altogether, these results firmly demonstrated that XMD8-92 and JWG-045 blocked RSL3-induced ferroptosis via a mechanism that did not involve the inhibition of ERK5 signalling.

### Analysis of the mechanism by which XMD8-92 mediate its anti-ferroptotic activity

**Fig. 7:**
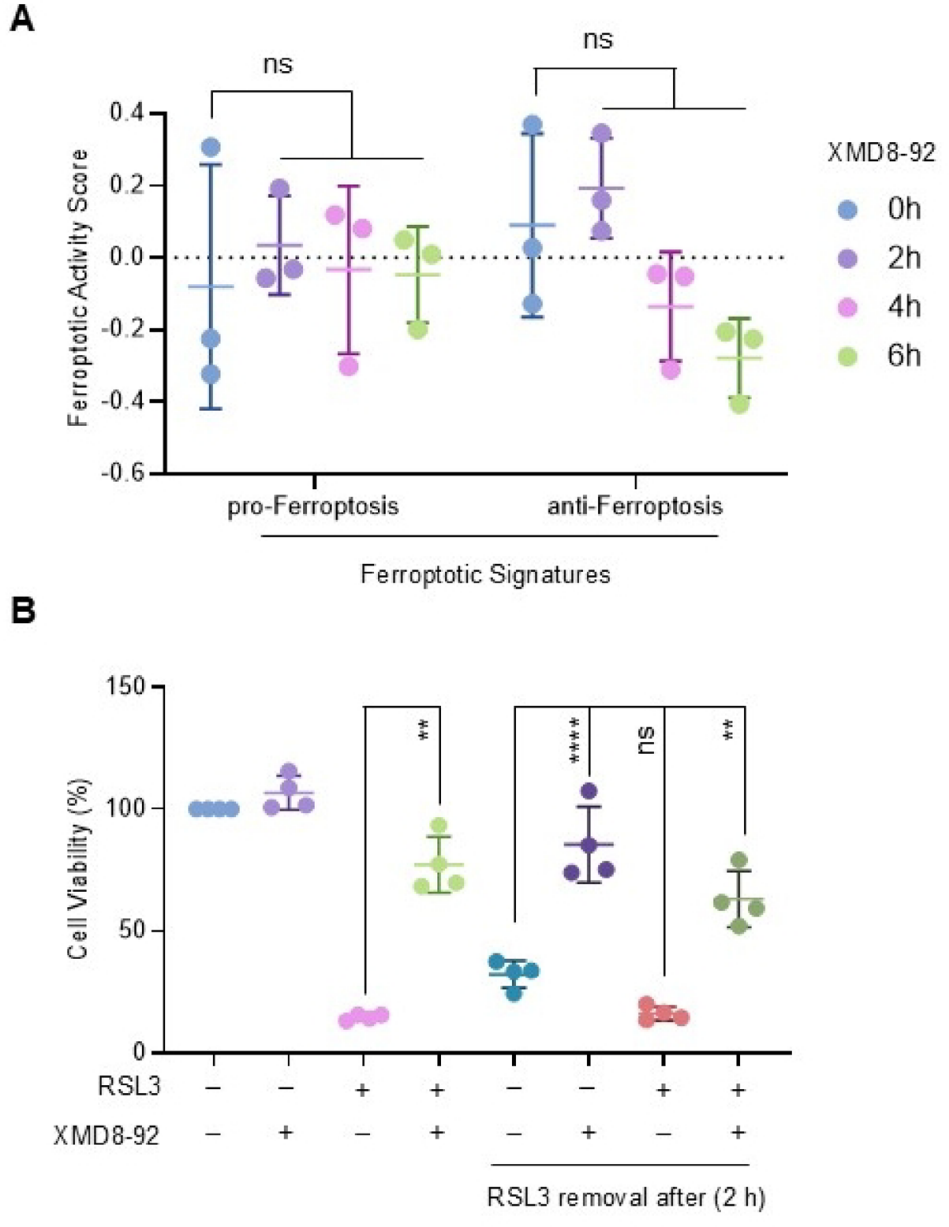
Effect of XMD8-92 on ferroptosis-associated activity scores and BT474 cell viability under ferroptotic stress. (**A**) RNA sequencing datasets were utilised to assess the level of ferroptotic activity in BT474 cells by GSVA of pro-ferroptotic and anti-ferroptotic gene sets from the FerrDb database, as described in our previous study^46^. One-way Anova was utilised to analyse statistical differences. (**B**) BT474 cells were pretreated with RSL3 (250 nM) for 2 h, prior to being incubated with XMD8-92 (5 µM) for 22 h. Alternatively, the cells were washed twice to remove RSL3 after 2 h and cultured for 22 h in fresh media containing XMD8-92 (5 µM), with or without RSL3. Mock treated cells were used as controls. Cell viability was quantified by CCK8 assay. The data are expressed as the mean percentage of cell viability ± SD (*n* = 4). Unpaired t-test was performed to determine statistical significance between RSL3 alone and RSL3 combined with XMD8-92. One-way ANOVA was performed to compare the effect of RSL3 withdrawal in the presence or absence of XMD8-92 and following RSL3 re-addition.

To further investigate the anti-ferroptotic effects of XMD8-92, we analysed RNA sequencing datasets from BT474 cells treated with the compound for 2, 4, or 6 h. Untreated cells (0 h) were used as controls. Principal component analysis (PCA) confirmed distinct clustering of the samples and high reproducibility across replicates (Supplementary Fig. S4A online). Differentially expressed genes (DEGs) in treated versus untreated cells were grouped into eight clusters based on temporal gene expression patterns (Supplementary Fig. S4B online). Heatmap visualisation of a subset of experimentally validated pro- and anti-ferroptotic genes from the FerrDb database^34^ revealed a gradual increase in anti-ferroptotic transcript levels and a concomitant decrease in pro-ferroptotic transcripts in BT474 cells over the time course of XMD8-92 treatment (Supplementary Fig. S4C online). However, pathway-level analysis using gene set variation analysis (GSVA) with the full FerrDb gene sets revealed no significant change in overall ferroptosis-related activity in the bulk RNA-sequencing data, but a modest downward trend in anti-ferroptotic scores (Fig. 7A). Notably, XMD8-92 treatment did not alter the level of *GPX4* transcript (Supplementary Fig. S5A online). This observation was corroborated at the protein level by immunoblot analysis (Supplementary Fig. S5B online).

**Fig. 8:**
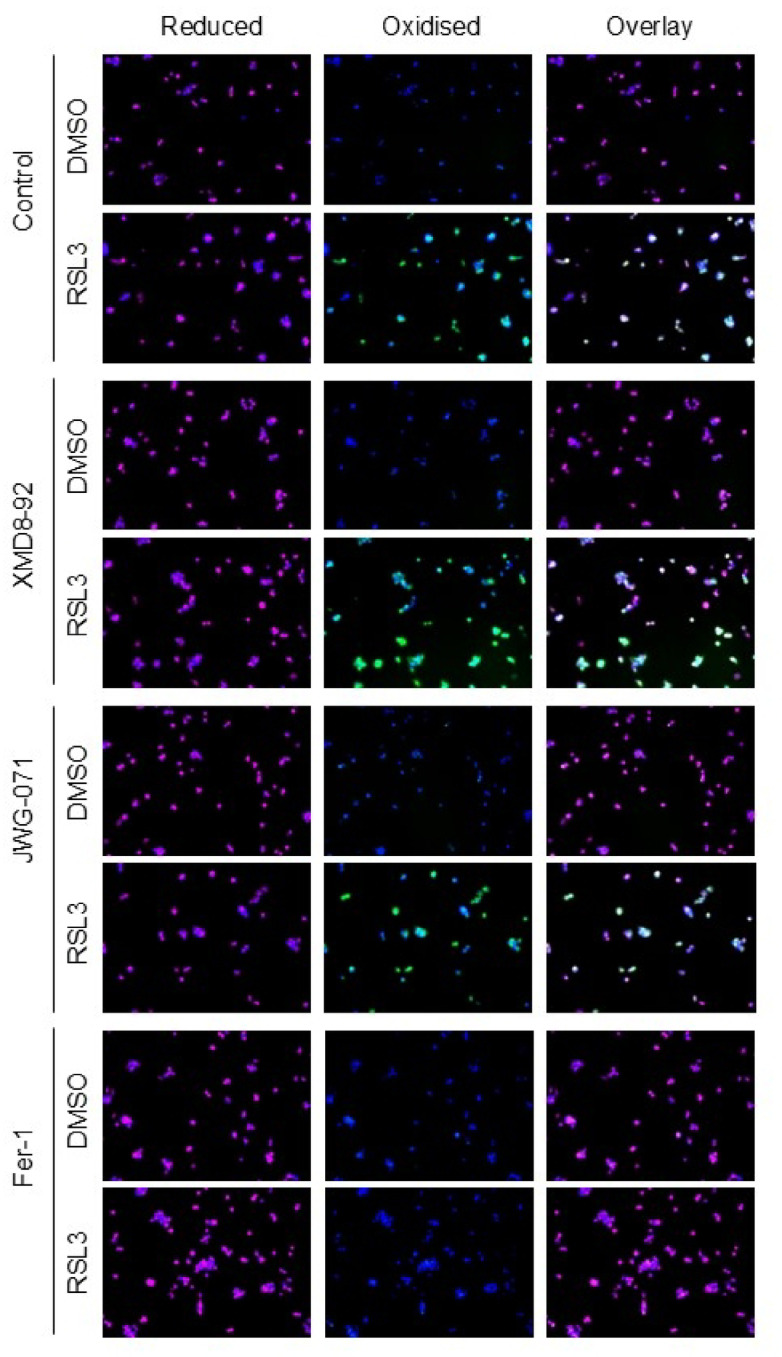
XMD8-92 failed to inhibit the RSL3-induced increase in lipid peroxidation in BT474 cells. BT474 cells were treated with RSL3 (250 nM), XMD8-92 (5 mM), JWG-071 (3 µM) or Ferrostatin-1 (Fer-1, 1 µM), as single agents or in combination for 2 h prior to being incubated for 10 min with BODIPY-C11^581/591^. Fluorescent signals were captured using a Thunder Widefield Microscope (Leica) to assess lipid peroxidation levels in cells.

To examine how XMD8-92 modulates the cellular response to RSL3 treatment, we performed BODIPY-C11 staining to quantify lipid peroxidation in BT474 cells treated with RSL3, XMD8-92, either alone or in combination. JWG-071-treated cells were included as negative controls. As expected, RSL3 induced lipid peroxidation, and this effect was not blocked by JWG-071 (Fig. 8). Surprisingly, XMD8-92 failed to inhibit the RSL3-induced increase in lipid peroxidation in BT474 cells (Fig. 8). In fact, XMD8-92 appeared to further enhanced lipid peroxidation in RSL3-treated cells, which is consistent with the observed trend toward reduced anti-ferroptotic GSVA scores over the time course of XMD8-92 treatment (Fig. 7A). Accumulation of lipid peroxides during ferroptosis compromises plasma membrane integrity, ultimately resulting in membrane rupture and cell death^12^. Accordingly, we found that a 2-hour pre-treatment with RSL3, followed by removal of the compound, was sufficient to commit cells to irreversible death, as cells failed to recover after withdrawal of the ferroptosis inducer (Fig. 7B). Remarkably, the addition of XMD8-92 following RSL3 removal partially restored cell viability.

Collectively, these results indicate that XMD8-92 does not prevent lipid peroxide formation but instead mitigates downstream membrane damage associated with ferroptotic cell death.

## Discussion

In this study, we explored a potential connection between ERK5 signalling and ferroptosis based on prior proteomic evidence identifying GPX4 as a binding partner of ERK5 [7] and bioinformatic analyses indicating that elevated *ERK5* mRNA expression is associated with increased sensitivity of cancer cells to GPX4 inhibitors (Fig. 1). Our experimental approach was based on utilising a panel of distinct inhibitors of ERK5 to rule out off target effects which led to controversial findings about the requirement of ERK5 in promoting cancer cell proliferation^35^. For example, the anti-proliferative effect of XMD8-92 observed in cancer cells which was comparable to that produced by ectopic expression of a dominant negative mutant of ERK5^17^, was later attributed to the off-target action of XMD8-92 on BRD4^25^. Similarly, we previously published that XMD8-92 at 25 µM blocked the ability of MDA-MB-231 cells to proliferate^19^. In this study, we showed that 24 h treatment with 5 µM XMD8-92 did not decrease MDA-MB-231 cell density (Figs. 2D and 4) and did not affect cell viability (Fig. 3F). Similarly, SUM159PT cells did not exhibit sensitivity to XMD8-92 treatment (Fig. 5B and C).

Although ERK5 is not generally required for cell proliferation and survival, evidence suggests that the pathway plays a role in growth and viability in a context-dependent manner. In particular, specific cellular backgrounds and environmental conditions appear to influence the dependency on ERK5 activity. For example, we demonstrated that HER2-expressing breast cancer cells, such as BT474, relied on ERK5 for sustained proliferation^6^. Here, we found that treatment with JWG-071 phenocopied the inhibitory effect of JWG-045 in BT474 cells following a 6-day incubation (Supplementary Fig. S2C online). Furthermore, like JWG-045^6^, JWG-071 restored trastuzumab sensitivity in BT474-derived trastuzumab-resistant cells to a level comparable to that seen with trastuzumab monotherapy in the parental line, thereby reinforcing the therapeutic relevance of ERK5 inhibition in HER2-positive breast cancers. However, by contrast with JWG-045 (Fig. 2C), short-term (24-hour) treatment with JWG-071, BAY-885, or BIX02189 had no effect on BT474 cell density (Fig. 3A and C).

In parallel to their growth inhibitory effect, we found that XMD8-92 and JWG-045 exhibited anti-ferroptotic activity which was not reproduced by JWG-071, BAY-885 or BIX02189. To investigate this further, we generated two different human-derived cancer cell lines, SUM159PT and HeLa, harbouring homozygous targeted deletion of *ERK5* by using CRISPR-mediated gene editing. The demonstration that XMD8-92 and JWG-045 protected ERK5-deficient cell lines from RSL3-induced ferroptosis to the same extent as the parental controls provided strong evidence that ERK5 was not involved in mediating the anti-ferroptotic effects of XMD8-92 and JWG-045 (Figs. 4, 5 and 6). We confirmed that BRD4 inhibitors did not display anti-ferroptotic activity, ruling this out as the target (Supplementary Fig. S3B online).

The apparent discrepancy between the observed changes in individual pro- and anti-ferroptotic transcripts (Supplementary Fig. S4 online) and the absence of a global alteration in ferroptotic pathway activity (Fig. 7A) likely reflects the limitations of transcriptional analyses in capturing functional ferroptosis regulation. Specifically, while XMD8-92 treatment induced modest shifts in specific ferroptosis-related genes, these changes may be insufficient to drive a coordinated pathway-level response detectable by GSVA. Additionally, ferroptosis is strongly regulated at the metabolic level, including lipid peroxidation dynamics. Interestingly, we found that XMD8-92 failed to prevent oxidative lipid damage, indicating that its cytoprotective effect is not mediated by inhibition of ferroptotic lipid peroxidation (Fig. 8). Furthermore, XMD8-92 was able to maintain cell viability even after the irreversible initiation of ferroptosis, confirming that its action occurs downstream of lipid peroxidation (Fig. 7B). Therefore, we propose that XMD8-92 may confer resistance to ferroptotic cell death by maintaining plasma membrane integrity, potentially through enhancing membrane repair mechanims^36,37^. However, this protection is likely temporary, as ongoing lipid peroxidation can eventually overwhelm membrane repair mechanisms and the damage to the plasma membrane becomes severe enough to render membrane rupture inevitable.

In conclusion, our study identified an off-target effect of the ERK5 inhibitors XMD8-92 and JWG-045 on ferroptosis, thereby further emphasising the need for careful evaluation of ERK5 inhibitors. Considering the paradoxical activation of ERK5 transcriptional activity by compounds of the XMD-series^28^, we suggest that JWG-071, BAY-885 and BIX02189 should be tested in parallel to investigate the phenotypic consequences of pharmacological inhibition of ERK5 signalling in future studies. To validate novel cellular functions, results of such experiments will need to be confirmed by using gene targeting approaches until novel MEK5/ERK5 inhibitors effective at nanomolar range concentrations become available. Furthermore, the apparent correlation between ERK5 and increased sensitivity of cancer cells to GPX4 inhibition revealed by bioinformatic data analyses is a reminder that correlative evidence does not prove causal relationship. In this case, we can assume that it is the mesenchymal nature of the cells which coincidently exhibit a high level of ERK5 that is responsible for increased sensitivity to ferroptotic-inducing agents^38,39^. Consequently, the functional significance of the binding interaction between ERK5 and GPX4 identified in our proteomic screen^7^ requires further investigation.

## Materials and methods

### Cell lines and cell culture

BT474, MDA-MB-231 and HeLa cells were originally purchased from the American Type Culture Collection (ATCC) and cultured in DMEM (Sigma-Aldrich #D6429) supplemented with 10% foetal bovine serum (FBS) (ThermoFisher Scientific #10500064). SUM159PT cells were obtained from Dr Sankari Nagarajan (The University of Manchester) and cultured in DMEM/F-12 (ThermoFisher Scientific #21331020), supplemented with 5% FBS, L-glutamine (2 mM, Sigma-Aldrich #G7513), insulin (5 µg/ml, Sigma-Aldrich #I9278) and hydrocortisone (1 µg/ml, Sigma-Aldrich #H0888). Cell authentication was not routinely conducted given that all cell lines were utilised for a maximum of 25 passages before thawing another aliquot of the same stock to maintain the original phenotype. Mycoplasma testing was routinely performed as described^40^.

### Cell seeding and treatments

Cells were seeded and allowed to adhere for 24 h before incubation with the following inhibitors: (i) ML210 (250 nM, Selleckchem #S0788) or RSL3 (250 nM or 2500 nM, Selleckchem #S8155) to induce ferroptosis; (ii) Ferrostatin-1 (1 µM, Selleckchem #S7243) to block ferroptosis; (iii) BIX02189 (5 µM, Selleckchem #S1531), XMD8-92 (5 µM, Selleckchem #S7525), JWG-045 [3 µM, a gift from Professor Nathanael Gray (Stanford University)], BAY-885 (5 µM, Selleckchem #S8896), or JWG-071 (3 µM, synthesised according to published protocols^18,24,26,41^) to inhibit ERK5 signalling. For combined treatments, cells were pre-incubated with the ERK5 inhibitors for 1 h before the addition of RSL3. Cell sensitivity to the treatments was performed in 24 well plates (5 × 10^4^ BT474 or 2.5 × 10^4^ MDA-MB-231 cells per well) or 12 well plates (2.5 × 10^4^ BT474, 1 × 10^4^ SUM159PT cells or 2 × 10^4^ HeLa cells per well) by measuring optical density (OD) at 570 nm after fixation in methanol and staining with crystal violet (Sigma-Aldrich) or by determining the number of viable cells by using the cell counting Kit-8 (CCK8; Tocris #7368). Alternatively, BT474 cell confluency was monitored in 24-well plates (5 × 10⁴ cells per well) using the IncuCyte SX5 system (Sartorius) equipped with a phase-contrast camera. Images were acquired every 2 h over a 20-hour period.

### Lipid peroxidation assay using BODIPY-C11

BODIPY-C11^581/591^ (MedChemExpress #HY-D1301,) was prepared as a 6 mM stock solution in fresh DMSO and stored at − 80 °C. For experiments, the stock was diluted in PBS to a final concentration of 2 µM. BT474 cells seeded at a density of 5 × 10^4^ cells per well in 24 well plates were washed once with PBS prior to adding the BODIPY-C11 solution. Plates were incubated at 37 °C for 10 min in the dark, washed once with PBS, and imaged using a Thunder Widefield Microscope (Leica). All steps were performed under minimal light exposure to prevent photobleaching.

### Generation of ERK5-knock-out cell lines by CRISPR-mediated gene editing

The co-expression construct comprising the “nickase” mutant Cas9 (D10A) sequence and dual gRNAs was purchased from VectorBuilder to target the exon 4 of *MAPK7* (sgRNA #1: 5’-cgcgctggtaccgtgcgccc-3’; sgRNA #2: 5’-atacacaggctattgacctc-3’). 3 × 10^5^ HeLa cells were seeded in 6-well plates and allowed to adhere overnight before transfection with jetPEI (Polyplus #101000053). 3 × 10^5^ SUM159PT cells were transfected using X-tremeGENE 9 (Roche #XTG9-RO) immediately after seeding while still in suspension. Cells were incubated for an additional 48 h before trypsinisation. Cell suspensions were adjusted to less than 10 cells/mL before seeding in 96-well plates with 100 µL per well. Single clones were expanded in 24-well plates before validation.

### Protein extraction and immunoblot analysis

Cells were lysed in RIPA buffer (Sigma-Aldrich #R0278) supplemented with protease and phosphatase inhibitors (ThermoFisher Scientific #87786 and #78420). Protein concentrations were quantified by DC™ protein assay (Bio-Rad #500 − 0112). Extracts (around 25 µg) were resolved by SDS-polyacrylamide gel electrophoresis (SDS-PAGE) and subjected to immunoblot analyses. The membranes were cut prior to hybridisation with antibodies to ERK5 (ABclonal #A3948) or β-tubulin (Abcam #ab6046). Immunocomplexes were detected by using the Odyssey^®^ CLx Imaging system with IRDye^®^ 800CW anti-Rabbit IgG secondary Antibody (LI-COR #926-32213).

### RNA-sequencing and bioinformatics analyses

BT474 cells were mock treated with DMSO or incubated with 5 µM XMD8-92 for 2, 4–6 h. RNA was extracted using the RNeasy Mini Kit (Qiagen #74104) and subjected to high throughput sequencing. Fastp (version 0.20.0) was utilised for trimming adaptors and removing low-quality reads to yield clean reads. Alignment of these clean reads to the human reference genome (hg38) was performed using the STAR (version 2.7.9a). The FeatureCounts (version 2.0) facilitated the acquisition of raw gene-level mRNA read counts, forming the basis of the mRNA expression profile. Annotation of the mRNA was conducted using the Ensembl GTF gene annotation database (version 104). DESeq2 package was used to assess fold changes and p-values between the two groups^42^. Mfuzz tool was used to evaluate the temporal patterns of gene expression over drug treatment^43^. GSVA tool was used to evaluate the EMT score based on the list of gene targets^44,45^. Gene expression and drug response correlation data were obtained from the CTRP (https://portals.broadinstitute.org/ctrp.v2.1/). Gene expression and CRISPR-screening based gene effect data were downloaded from DepMap portal (Data version. 23Q4, https://depmap.org/portal/).

### Statistical analyses

Data were plotted as means of at least 3 biological repeats and error bars indicate standard deviations (SD). The GraphPad Prism 10.0 software was employed for all statistical analyses using unpaired t-test for direct comparison of two conditions or one-way ANOVA for comparison of multiple conditions. Differences were considered statistically significant when the p value was less than 0.05. *p ≤ 0.05; **p ≤ 0.01; ***p ≤ 0.001; ****p ≤ 0.0001, ns = non-significant.

## Supplementary Information

Below is the link to the electronic supplementary material.

Supplementary Material 1

## Data Availability

All data and materials supporting the findings of this study are available within the article and its supplementary information files online or from the corresponding author upon reasonable request. RNA sequencing datasets of BT474 cells incubated for various times with DMSO or with XMD8-92 are available in the GEO repository with the accession number GSE302031.

## References

[1] Nithianandarajah-Jones G. N. et al. ERK5: structure, regulation and function. *Cell. Signal.* **24**, 2187–2196 (2012). 22800864 10.1016/j.cellsig.2012.07.007

[2] Stecca, B. & Rovida, E. Impact of ERK5 on the Hallmarks of Cancer. *Int. J. Mol. Sci.* **20**, 1426 (2019). 30901834 10.3390/ijms20061426 PMC6471124

[3] Montero, J. C. et al. Expression of Erk5 in early stage breast cancer and association with disease free survival identifies this kinase as a potential therapeutic target. *PLoS One*. **4**, e5565 (2009). 19440538 10.1371/journal.pone.0005565 PMC2678256

[4] Ortiz-Ruiz, M. J. et al. Therapeutic potential of ERK5 targeting in triple negative breast cancer. *Oncotarget* **5**, 11308–11318 (2014). 25350956 10.18632/oncotarget.2324 PMC4294347

[5] Xu, Q. et al. The extracellular-regulated protein kinase 5 (ERK5) enhances metastatic burden in triple-negative breast cancer through focal adhesion protein kinase (FAK)-mediated regulation of cell adhesion. *Oncogene* **40**, 3929–3941 (2021). 33981002 10.1038/s41388-021-01798-2 PMC8195737

[6] Zhang, J. et al. Inhibiting ERK5 overcomes breast cancer resistance to anti-HER2 therapy by targeting the G1/S cell cycle transition. *Cancer Res. Commun.* **2**, 131–145 (2022). 36466034 10.1158/2767-9764.CRC-21-0089 PMC7613885

[7] Pearson, A. J. et al. Discovery of a Gatekeeper Residue in the C-Terminal Tail of the Extracellular Signal-Regulated Protein Kinase 5 (ERK5). *Int. J. Mol. Sci.* **21**, 929 (2020). 32023819 10.3390/ijms21030929 PMC7037328

[8] Brigelius-Flohé, R. & Maiorino, M. Glutathione peroxidases. *Biochim. Biophys. Acta*. **1830**, 3289–3303 (2013). 23201771 10.1016/j.bbagen.2012.11.020

[9] Weïwer, M. et al. Development of small-molecule probes that selectively kill cells induced to express mutant RAS. *Bioorg. Med. Chem. Lett.* **22**, 1822–1826 (2012). 22297109 10.1016/j.bmcl.2011.09.047 PMC3528973

[10] Yang, W. S. et al. Regulation of ferroptotic cancer cell death by GPX4. *Cell* **156**, 317–331 (2014). 24439385 10.1016/j.cell.2013.12.010 PMC4076414

[11] Eaton, J. K. et al. Selective covalent targeting of GPX4 using masked nitrile-oxide electrophiles. *Nat. Chem. Biol.* **16**, 497–506 (2020). 32231343 10.1038/s41589-020-0501-5 PMC7251976

[12] Jiang, X., Stockwell, B. R. & Conrad, M. Ferroptosis: mechanisms, biology and role in disease. *Nat. Rev. Mol. Cell. Biol.* **22**, 266–282 (2021). 33495651 10.1038/s41580-020-00324-8 PMC8142022

[13] Viswanathan, V. S. et al. Dependency of a therapy-resistant state of cancer cells on a lipid peroxidase pathway. *Nature* **547**, 453–457 (2017). 28678785 10.1038/nature23007 PMC5667900

[14] Hangauer, M. J. et al. Drug-tolerant persister cancer cells are vulnerable to GPX4 inhibition. *Nature* **551**, 247–250 (2017). 29088702 10.1038/nature24297 PMC5933935

[15] Schwab, A. et al. Zeb1 mediates EMT/plasticity-associated ferroptosis sensitivity in cancer cells by regulating lipogenic enzyme expression and phospholipid composition. *Nat. Cell. Biol.* **26**, 1470–1481 (2024). 39009641 10.1038/s41556-024-01464-1 PMC11392809

[16] Pereira, D. M. & Rodrigues, C. M. P. Targeted Avenues for Cancer Treatment: The MEK5-ERK5 Signaling Pathway. *Trends Mol. Med.* **26**, 394–407 (2020). 32277933 10.1016/j.molmed.2020.01.006

[17] Yang, Q. et al. Pharmacological inhibition of BMK1 suppresses tumor growth through promyelocytic leukemia protein. *Cancer Cell.* **18**, 258–267 (2010). 20832753 10.1016/j.ccr.2010.08.008 PMC2939729

[18] Deng, X. et al. Discovery of a benzo[e]pyrimido-[5,4-b][1,4]diazepin-6(11H)-one as a Potent and Selective Inhibitor of Big MAP Kinase 1. *ACS Med. Chem. Lett.* **2**, 195–200 (2011). 21412406 10.1021/ml100304b PMC3055678

[19] Perez-Madrigal, D., Finegan, K. G., Paramo, B. & Tournier, C. The extracellular-regulated protein kinase 5 (ERK5) promotes cell proliferation through the down-regulation of inhibitors of cyclin dependent protein kinases (CDKs). *Cell. Signal.* **24**, 2360–2368 (2012). 22917534 10.1016/j.cellsig.2012.08.001

[20] Yang, Q. et al. BMK1 is involved in the regulation of p53 through disrupting the PML-MDM2 interaction. *Oncogene* **32**, 3156–3164 (2013). 22869143 10.1038/onc.2012.332 PMC3493705

[21] Rovida, E. et al. The mitogen-activated protein kinase ERK5 regulates the development and growth of hepatocellular carcinoma. *Gut* **64**, 1454–1465 (2015). 25183205 10.1136/gutjnl-2014-306761

[22] Finegan, K. G. et al. ERK5 is a critical mediator of inflammation-driven cancer. *Cancer Res.* **75**, 742–753 (2015). 25649771 10.1158/0008-5472.CAN-13-3043 PMC4333217

[23] Wilhelmsen, K. et al. Extracellular signal-regulated kinase 5 promotes acute cellular and systemic inflammation. *Sci. Signal.* **8**, ra86 (2015). 26307013 10.1126/scisignal.aaa3206 PMC5734625

[24] Williams, C. A. et al. Erk5 Is a Key Regulator of Naive-Primed Transition and Embryonic Stem Cell Identity. *Cell. Rep.* **16**, 1820–1828 (2016). 27498864 10.1016/j.celrep.2016.07.033 PMC4987282

[25] Lin, E. C. et al. ERK5 kinase activity is dispensable for cellular immune response and proliferation. *Proc. Natl. Acad. Sci. U S A*. **113**, 11865–11870 (2016). 27679845 10.1073/pnas.1609019113 PMC5081620

[26] Deng, X. et al. Structural determinants for ERK5 (MAPK7) and leucine rich repeat kinase 2 activities of benzo[e]pyrimido-[5,4-b]diazepine-6(11H)-ones. *Eur. J. Med. Chem.* **70**, 758–767 (2013). 24239623 10.1016/j.ejmech.2013.10.052 PMC3914206

[27] Sureban, S. M. et al. XMD8-92 inhibits pancreatic tumor xenograft growth via a DCLK1-dependent mechanism. *Cancer Lett.* **351**, 151–161 (2014). 24880079 10.1016/j.canlet.2014.05.011

[28] Lochhead, P. A. et al. Paradoxical activation of the protein kinase-transcription factor ERK5 by ERK5 kinase inhibitors. *Nat. Commun.* **11**, 1383 (2020). 32170057 10.1038/s41467-020-15031-3 PMC7069993

[29] Wang, J. et al. Structural and Atropisomeric Factors Governing the Selectivity of Pyrimido-benzodiazipinones as Inhibitors of Kinases and Bromodomains. *ACS Chem. Biol.* **13**, 2438–2448 (2018). 30102854 10.1021/acschembio.7b00638 PMC6333101

[30] Nguyen, D. et al. Discovery and Characterization of the Potent and Highly Selective (Piperidin-4-yl)pyrido[3,2- d]pyrimidine Based in Vitro Probe BAY-885 for the Kinase ERK5. *J. Med. Chem.* **62**, 928–940 (2019). 30563338 10.1021/acs.jmedchem.8b01606

[31] Zhao, W. et al. Inhibition of MEK5/ERK5 signaling overcomes acquired resistance to the third generation EGFR inhibitor, osimertinib, via enhancing Bim-dependent apoptosis. *Cancer Lett.* **519**, 141–149 (2021). 34245854 10.1016/j.canlet.2021.07.007

[32] Tatake, R. J. et al. Identification of pharmacological inhibitors of the MEK5/ERK5 pathway. *Biochem. Biophys. Res. Commun.* **377**, 120–125 (2008). 18834865 10.1016/j.bbrc.2008.09.087

[33] Flanagan, L., Van Weelden, K., Ammerman, C., Ethier, S. P. & Welsh, J. SUM-159PT cells: a novel estrogen independent human breast cancer model system. *Breast Cancer Res. Treat.* **58**, 193–204 (1999). 10718481 10.1023/a:1006331716981

[34] Zhou, N. et al. FerrDb V2: update of the manually curated database of ferroptosis regulators and ferroptosis-disease associations. *Nucleic Acids Res.* **51**, D571–D582 (2023). 36305834 10.1093/nar/gkac935 PMC9825716

[35] Lochhead, P. A., Gilley, R. & Cook, S. J. ERK5 and its role in tumour development. *Biochem. Soc. Trans.* **40**, 251–256 (2012). 22260700 10.1042/BST20110663

[36] Dai, E., Meng, L., Kang, R., Wang, X. & Tang, D. ESCRT-III-dependent membrane repair blocks ferroptosis. *Biochem. Biophys. Res. Commun.* **522**, 415–421 (2020). 31761326 10.1016/j.bbrc.2019.11.110 PMC6957708

[37] Pedrera, L. et al. García-Sáez AJ. Ferroptotic pores induce Ca^2+^ fluxes and ESCRT-III activation to modulate cell death kinetics. *Cell. Death Differ.* **28**, 1644–1657 (2021). 33335287 10.1038/s41418-020-00691-x PMC8167089

[38] Pavan, S. et al. A kinome-wide high-content siRNA screen identifies MEK5-ERK5 signaling as critical for breast cancer cell EMT and metastasis. *Oncogene* **37**, 4197–4213 (2018). 29713055 10.1038/s41388-018-0270-8

[39] Bhatt, A. B. et al. Diverse and converging roles of ERK1/2 and ERK5 pathways on mesenchymal to epithelial transition in breast cancer. *Transl Oncol.* **14**, 101046 (2021). 33761370 10.1016/j.tranon.2021.101046 PMC8020482

[40] Young, L., Sung, J., Stacey, G. & Masters, J. R. Detection of Mycoplasma in cell cultures. *Nat. Protoc.* **5**, 929–934 (2010). 20431538 10.1038/nprot.2010.43

[41] Miduturu, C. V. et al. High-throughput kinase profiling: a more efficient approach toward the discovery of new kinase inhibitors. *Chem. Biol.* **18**, 868–879 (2011). 21802008 10.1016/j.chembiol.2011.05.010 PMC3171802

[42] Love, M. I., Huber, W. & Anders, S. Moderated estimation of fold change and dispersion for RNA-seq data with DESeq2. *Genome Biol.* **15**, 550 (2014). 25516281 10.1186/s13059-014-0550-8 PMC4302049

[43] Kumar, L. & Futschik, E. Mfuzz: a software package for soft clustering of microarray data. *Bioinformation* **2**, 5–7 (2007). 18084642 10.6026/97320630002005 PMC2139991

[44] Hänzelmann, S., Castelo, R. & Guinney, J. GSVA: gene set variation analysis for microarray and RNA-seq data. *BMC Bioinform.* **14**, 7 (2013). 10.1186/1471-2105-14-7 PMC3618321 23323831

[45] Byers, L. A. et al. An epithelial-mesenchymal transition gene signature predicts resistance to EGFR and PI3K inhibitors and identifies Axl as a therapeutic target for overcoming EGFR inhibitor resistance. *Clin. Cancer Res.* **19**, 279–290 (2013). 23091115 10.1158/1078-0432.CCR-12-1558 PMC3567921

[46] Zhang, W. et al. NRF2-mediated persistent adaptation of oesophageal adenocarcinoma cells to HER2 inhibition. *Oncogene* **44**, 2929–2941 (2025). 40473905 10.1038/s41388-025-03459-0 PMC12336050

