# Supplementary material for: XMD8-92 and JWG-045 exhibit anti-ferroptotic activities, independently of inhibiting ERK5
Source: Sci Rep. 2026 Feb 28;16:11337. doi: 10.1038/s41598-026-42079-w (PMC13049074; doi:10.1038/s41598-026-42079-w)
Supplement: Supplementary file 1 — Supplementary Material 1 [file 41598_2026_42079_MOESM1_ESM.pdf]

Supplementary Information for:

XMD8-92 and JWG-045 exhibit anti-ferroptotic activities, independently of inhibiting ERK5

Wei Zhang, Karmern Kan, Aidan B Pidd, Gala Konteva, Weitao Xiao, Adam J Pearson, Zejia Song, Qiuping Xu, Sam Butterworth, Alan J Whitmarsh and Cathy Tournier

Running title: XMD8-92 and JWG-045 exhibit anti-ferroptotic effects

Supplementary Figure legends

Supplementary Figure S1. Chemical structures of MEK5 and ERK5 inhibitors.

This figure shows the chemical structures of the small-molecule inhibitors targeting MEK5 and ERK5 pathways investigated in this study. ERK5 inhibitors include XMD8-92, JWG-045, JWG-071, and BAY-885, while BIX02189 is shown as a representative MEK5 inhibitor. These structures are presented to highlight differences in scaffold and potential off-target activity that may contribute to their distinct biological effects.

Supplementary Figure S2. Pharmacological inhibition of ERK5 increases the therapeutic sensitivity of both sensitive and resistant BT474 cell lines to trastuzumab.

A and **B** Immunoblot analysis of ERK5 expression in BT474 cells (A) and MDA-MB-231 cells (B) treated with XMD8-92 (5 μM), JWG-045 (J-045; 3 μM), JWG-071 (J-071; 3 μM), BAY-885 (B-885; 5 μM) or BIX-02189 (BIX; 5 μM) for 1 h, followed by EGF (10 ng/mL) stimulation for 10 minutes where indicated. Mock treated cells with DMSO were used as controls. β-tubulin expression was used as loading control. Similar results were obtained in two independent experiments. The arrow indicates phosphorylated ERK5. **C** Parental and trastuzumab-resistant BT474 cells were treated with JWG-045 (3 μM), JWG-071 (3 μM), or trastuzumab (10 μg/mL), as single agents or in combination, for 6 days, followed by crystal violet staining and quantification. Mock treated cells with DMSO were used as controls. The data are expressed as mean percentages of cell density ± SD (n = 3). One-way ANOVA was utilised to compare the effect of trastuzumab versus trastuzumab combined with ERK5 inhibitor treatments, or the effect of DMSO versus ERK5 inhibitor treatments alone; unpaired t test was utilised to analyse statistical differences between the effects of JWG-045 versus JWG-071.

Supplementary Figure S3. BRD4 is not involved in mediating the distinct effects of ERK5 inhibitors on ferroptosis

A BT474 cells were treated with XMD8-92 (5 μM), JWG-045 (3 μM), JWG-071 (3 μM), BAY-885 (5 μM), Ferrostatin-1 (Fer-1, 1 μM), or ML210 (250 nM), as single agents or in combination, for 24 hours. Mock treated cells with DMSO were used as controls. Cell density was quantified by crystal violet staining. The data are expressed as mean percentages of cell density ± SD (n = 3). **B** BT474 cells were treated with JWG-071 (3 μM), JQ1 (1 μM) or Birabresib (1 μM), as single agents or in combination for 2 hours before adding RSL3 (250 nM) for another 24 hours. Mock treated cells with DMSO were used as controls. Cell density was quantified by crystal violet staining. The data are expressed as mean percentages of cell density ± SD (n = 3). One-way ANOVA was performed for statistical analyses (A and B).

Supplementary Figure S4. ERK5 inhibition by XMD8-92 influences the expression of a subset of experimentally validated pro- and anti-ferroptotic genes from the FerrDb database.

BT474 cells were mock treated with DMSO or incubated with 5 μM XMD8-92 for 2 h, 4 h or 6 h. **A** PCA plot of RNA-sequencing datasets of BT474 cells mock treated with DMSO or incubated with 5 μM XMD8-92 for 2 h, 4 h or 6 h (n = 3). The clustering indicates the reproducibly of samples. **B** DEGs (vs. DMSO, adjusted $p < 0.01$, logFC cut-off value 0.585) were selected for Mfuzz time-course analysis. Genes were grouped into 8 clusters, according to their expression pattern over the time course of XMD8-92 treatment. **C** Heatmap plot representing the transcriptional expression profile of anti- and pro-ferroptotic genes over the time course of XMD8-92 treatment. Genes in clusters 1 to 3 were intersected with validated anti-ferroptotic genes, while genes in clusters 4 to 7 were intersected with validated pro-ferroptotic genes from FerrDb database. Cluster 8 comprised both anti- and pro-ferroptotic

genes; warm colours (shade of red) signify high expression and cool colours (shades of blue) low expression based on row z-scores. Each column represents a sample.

Supplementary Figure S5. XMD8-92 incubation does not alter the level of GPX4 expression in breast cancer cells.

A Detection of *GPX4* transcript levels in BT474 cells treated with XMD8-92 (5 μM) for the indicated time by RNAseq (n = 3). Unpaired t-test was utilised to analyse statistical differences. **B** Immunoblot analysis of GPX4 (Proteintech, #67763-1-Ig) expression in BT474 cells pre-treated with XMD8-92 (XMD8; 5 μM) or JWG-071 (J-071; 3 μM) for 1 h, prior to being incubated with RSL3 (250 nM) for 2 h. Alternatively, cells were treated with XMD8-92 or JWG-071 alone for 24 h. Mock treated cells with DMSO were used as controls. β-tubulin expression was used as loading control. Fluorescent signals were analysed by using the Odessey system. Intensities were normalised to that of β-tubulin and the level of GPX4 is presented as fold of DMSO control (−RSL3, 2 h). The data correspond to the means ± SD (n = 4), and statistical significance was determined by one-way ANOVA.

Supplementary Figure S6. Original replicate images acquired using the Odyssey® CLx Imaging System and used for Figure 4A are shown with the blot edges and the dates on which each immunoblot was performed.

Supplementary Figure S7. Original replicate images acquired using the Odyssey® CLx Imaging System and used for Figure 5A are shown with the blot edges and the dates on which each immunoblot was performed.

Supplementary Figure S8. Original replicate images acquired using the Odyssey® CLx

Imaging System and used for Figure 6A are shown with the blot edges and the dates on which each immunoblot was performed.

Supplementary Figure S9. Original replicate images acquired using the Odyssey® CLx Imaging System and used for Supplementary Figures S2A and S2B are shown with the blot edges and the dates on which each immunoblot was performed.

Supplementary Figure S10. Original replicate images acquired using the Odyssey® CLx Imaging System and used for Supplementary Figure S5B are shown with the blot edges and the dates on which each immunoblot was performed.

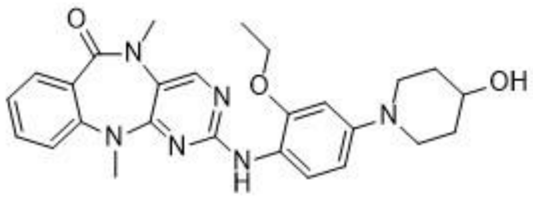

XMD8-92

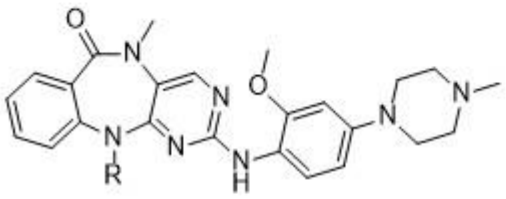

JWG-045, R 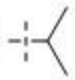

JWG-071, R 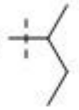

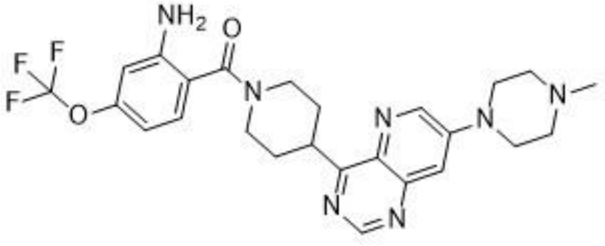

BAY-885

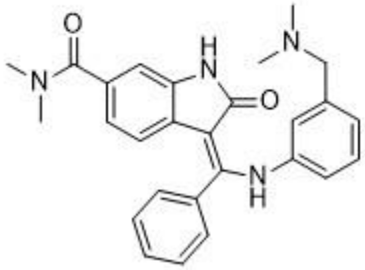

BIX02189

Supplementary Figure S1

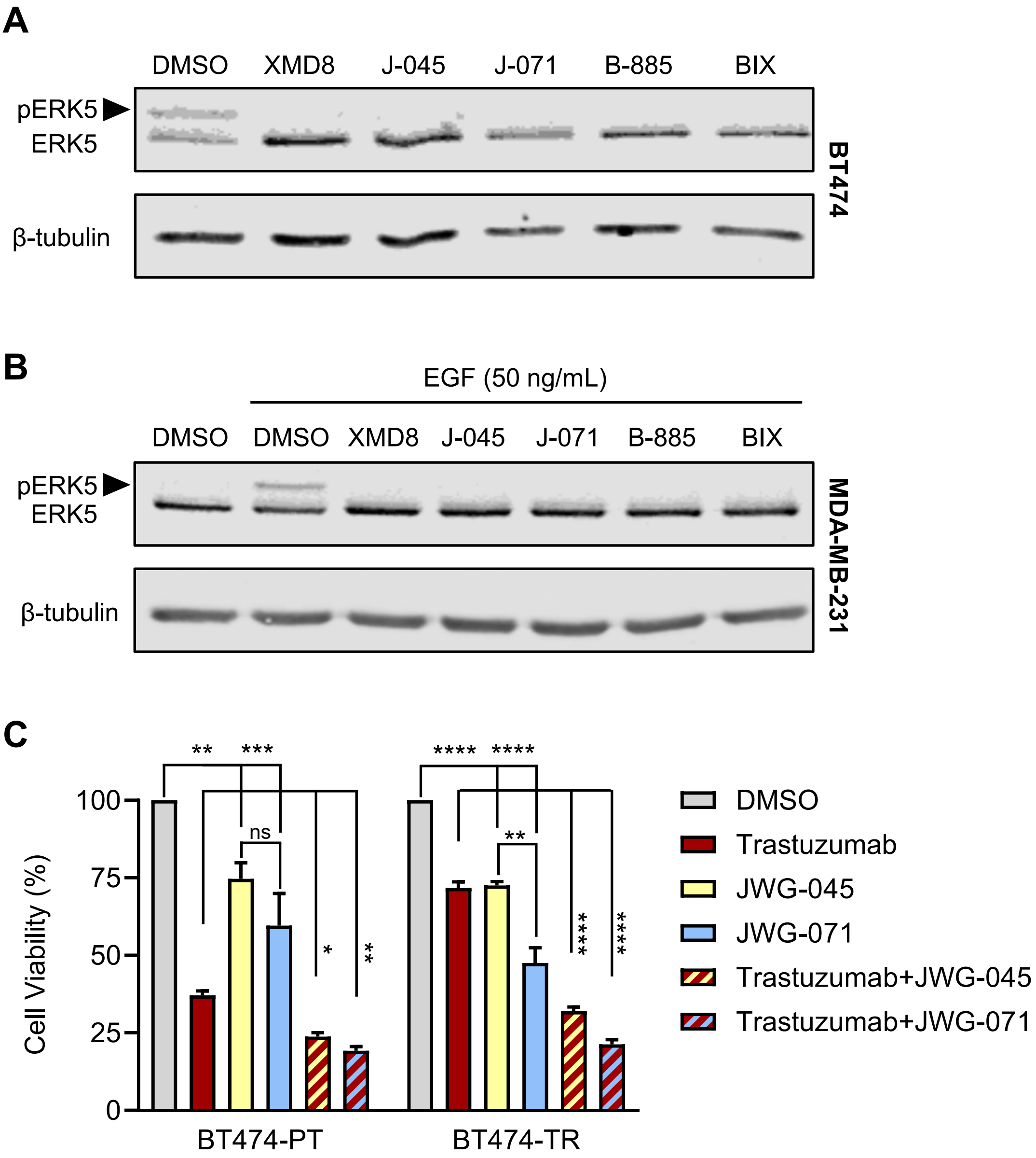

Supplementary Figure S2

BT474

A

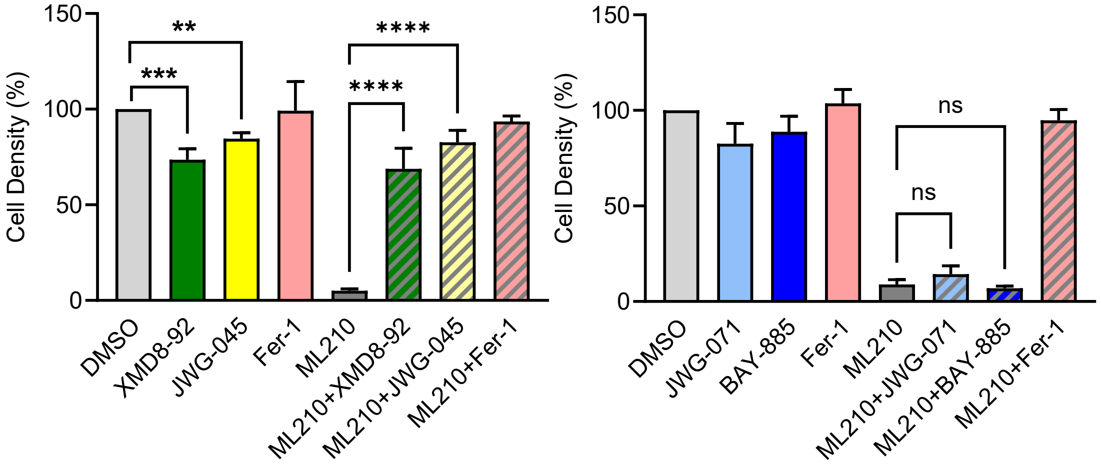

B

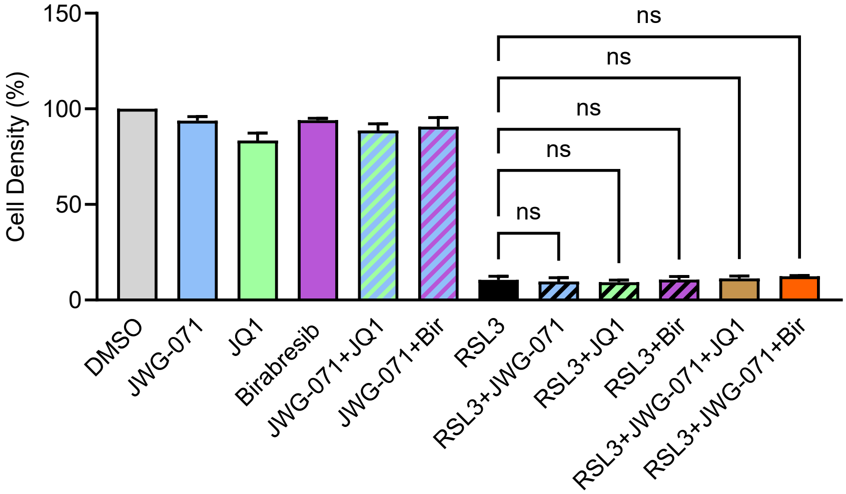

Supplementary Figure S3

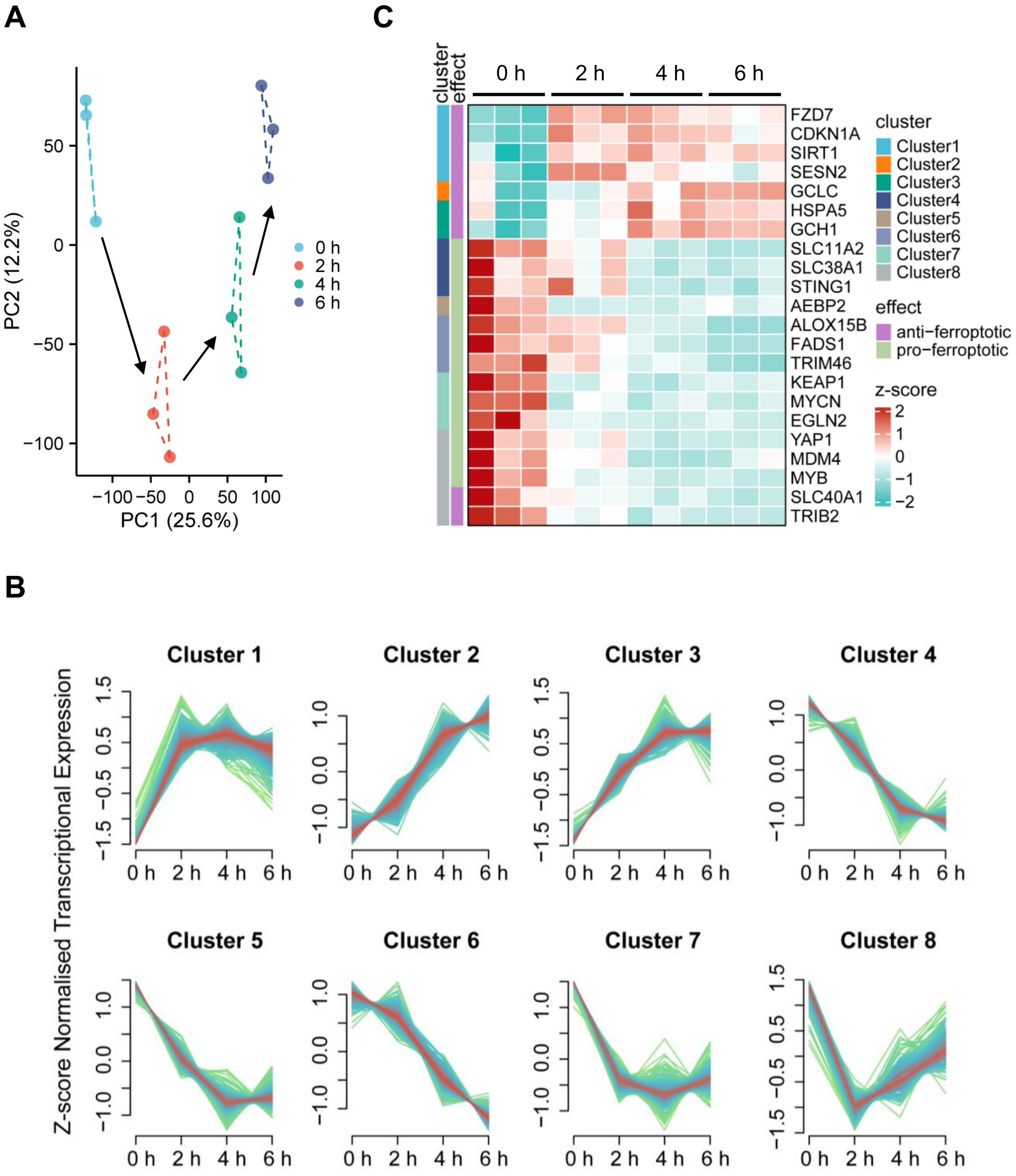

Supplementary Figure S4

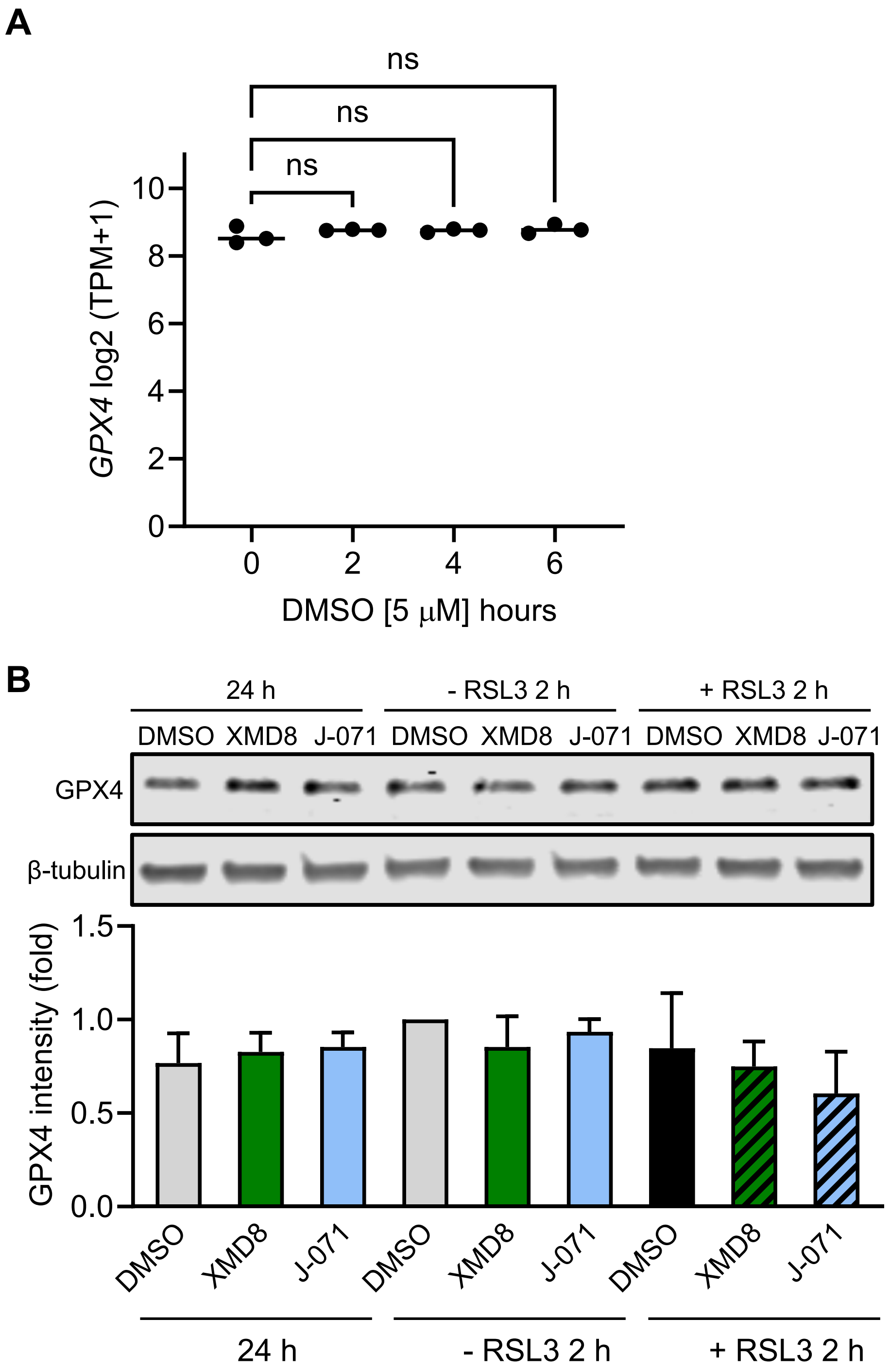

Supplementary Figure S5

MDA-MB-231

4th March 2023

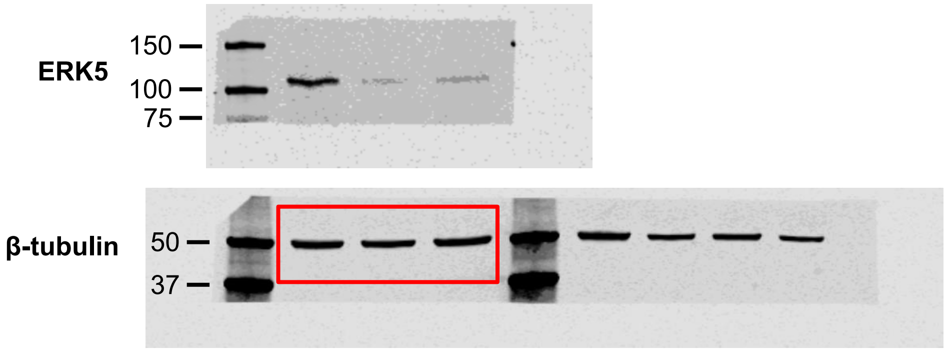

27th February 2023

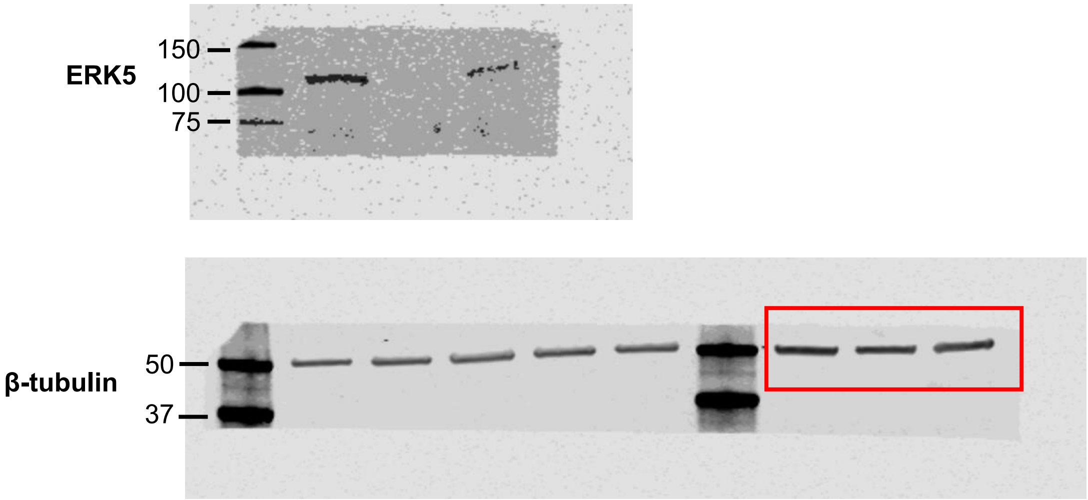

Supplementary Figure S6

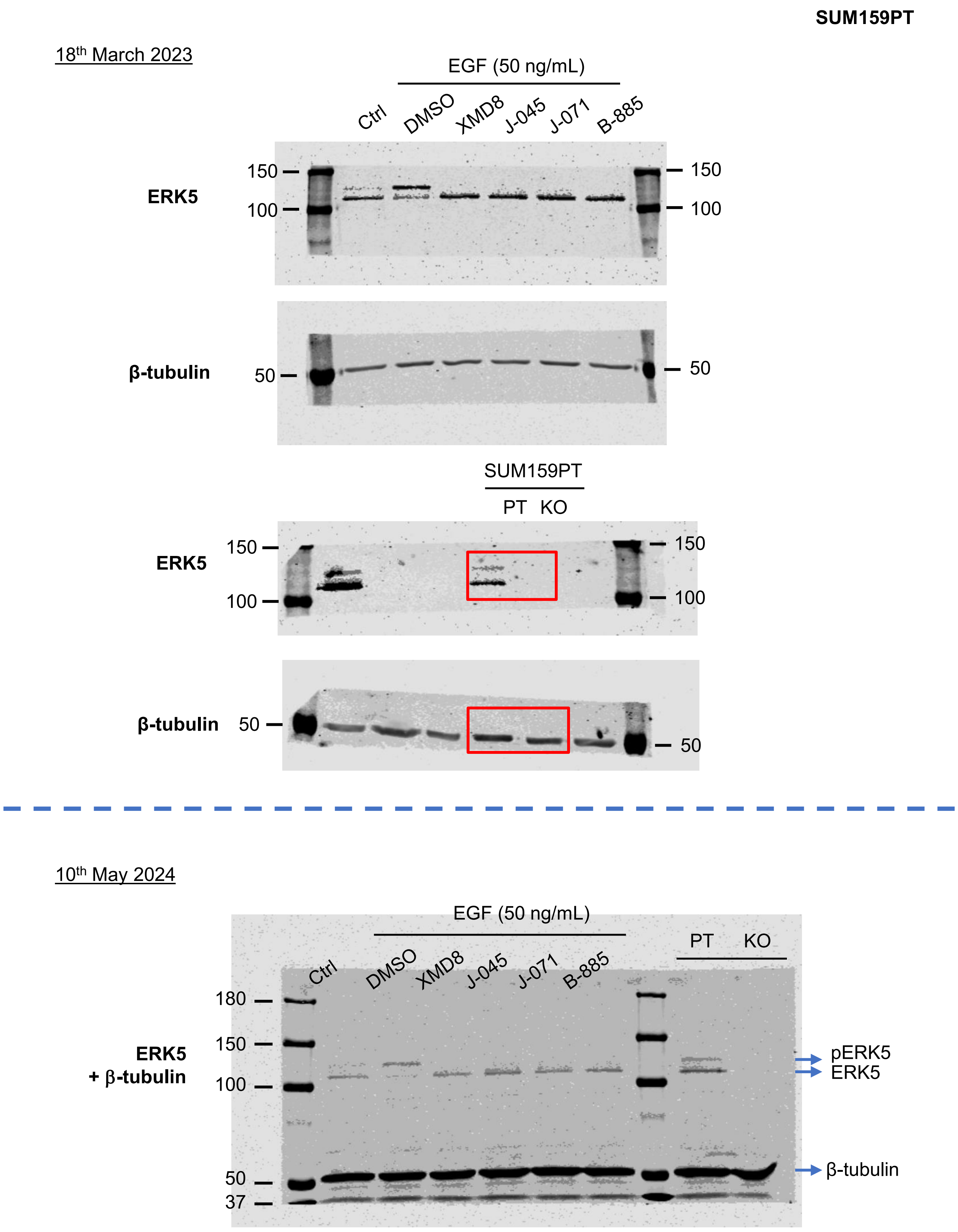

Supplementary Figure S7

HeLa

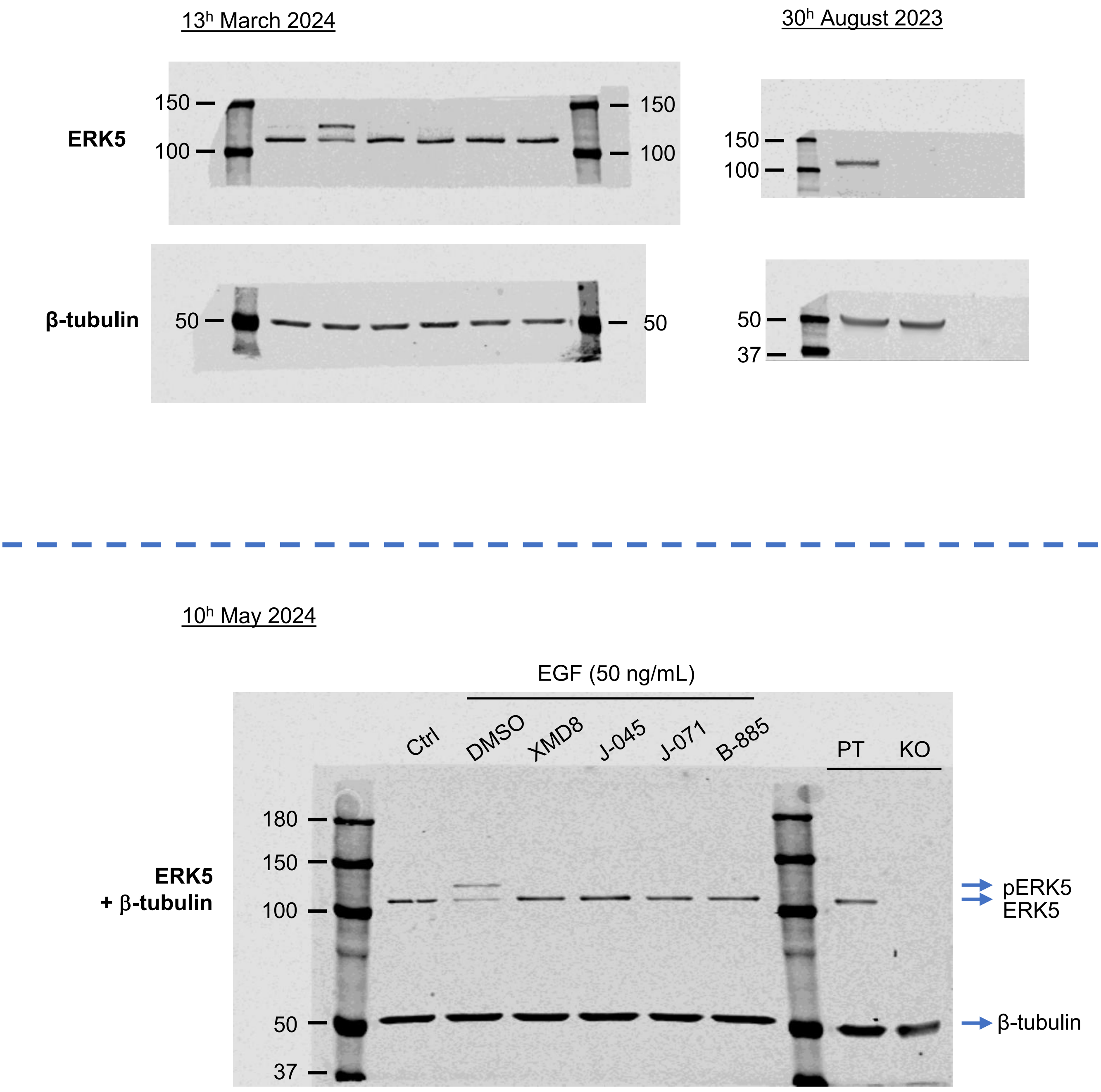

Supplementary Figure S8

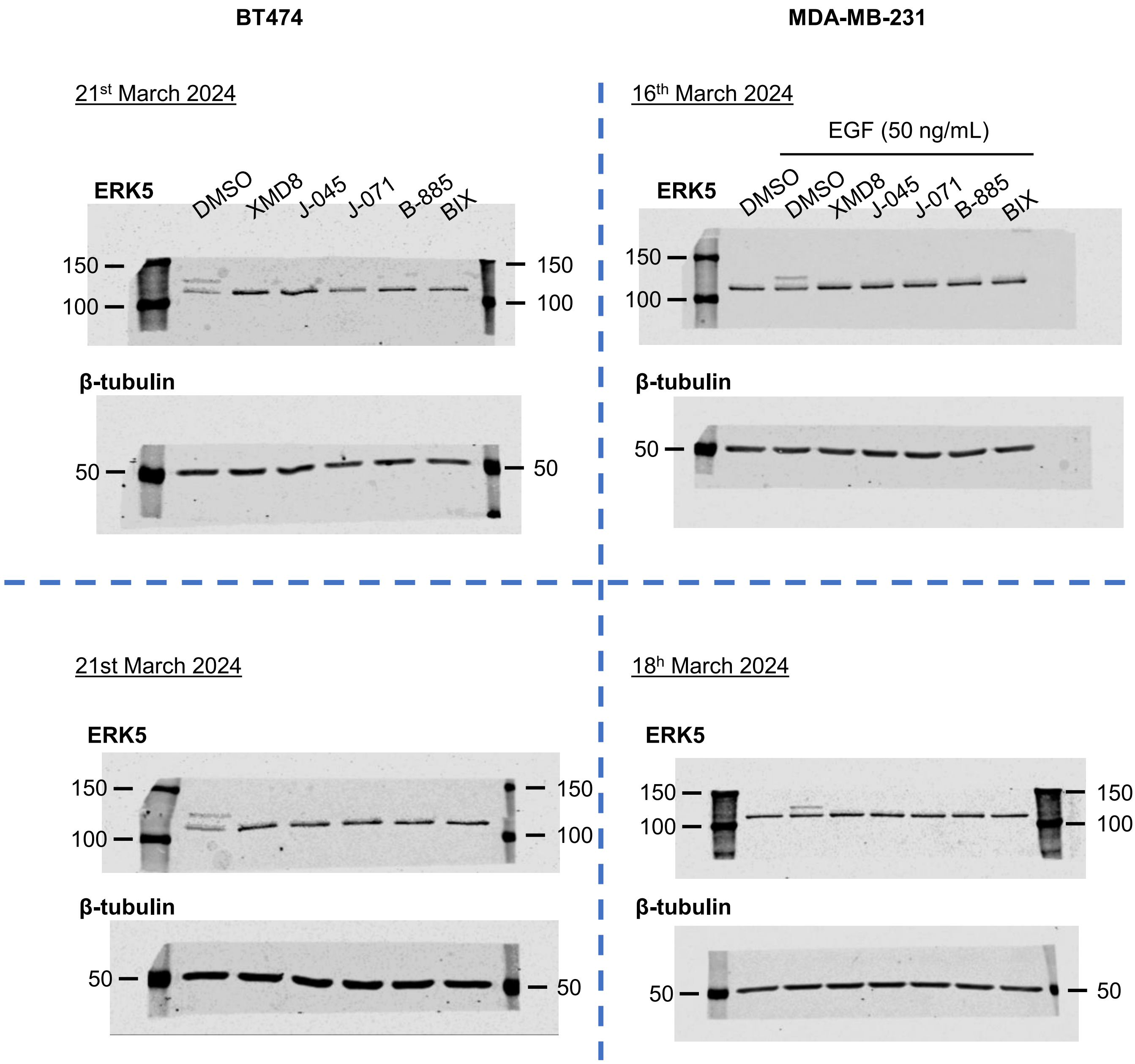

Supplementary Figure S9

BT474

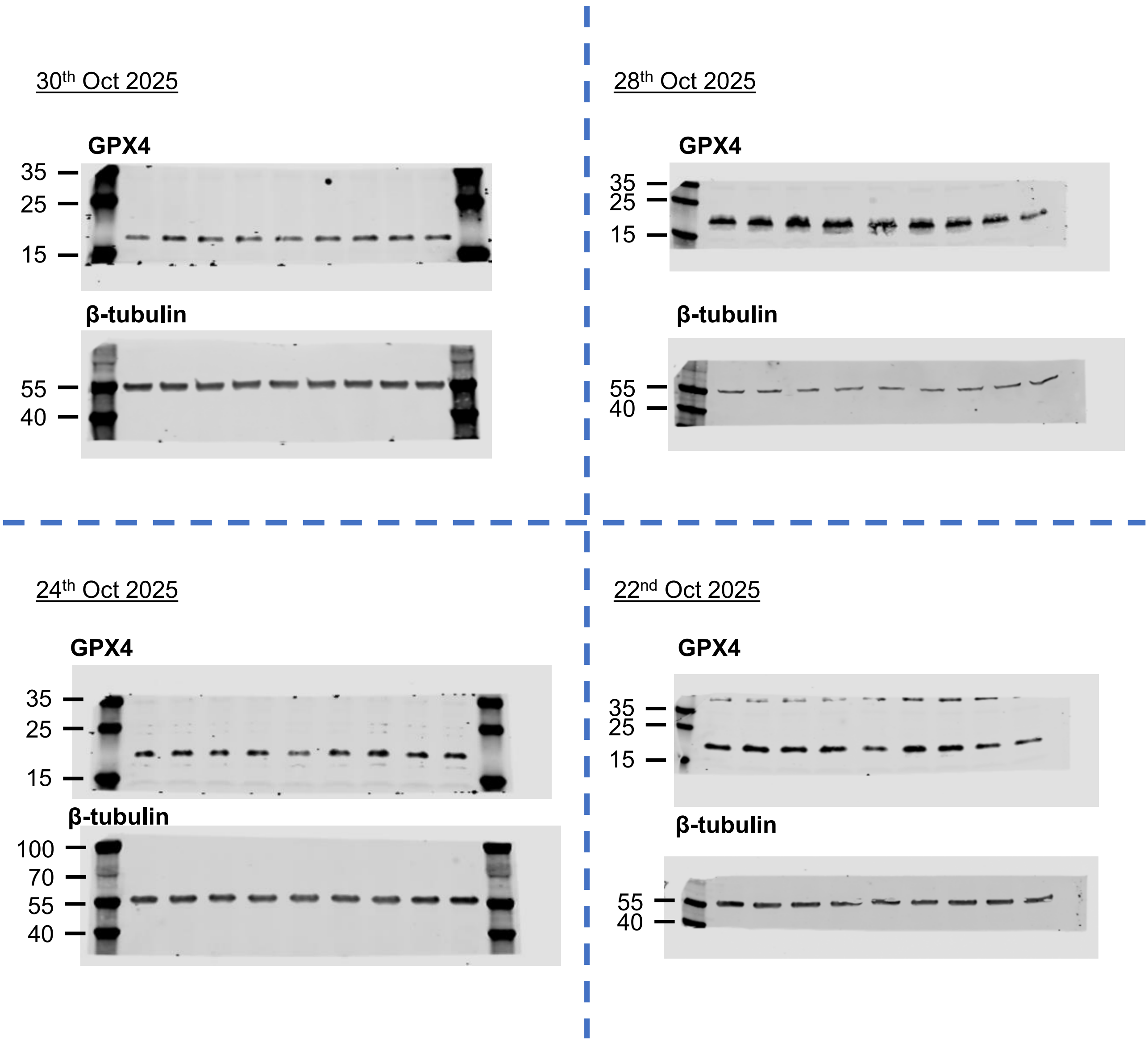

Supplementary Figure S10
